# Vorticity-induced short-range ordering for heat insulation in anisotropically bonded CsAg_2_I_3_

**DOI:** 10.1038/s41467-026-76794-9

**Published:** 2026-08-21

**Authors:** Qingyu Bai, Long Yang, Zhiwei Chen, Changyuan Li, Ruihuan Cheng, Mingfang Shu, Jinfeng Zhu, Ziyue Liu, Yuyan Yang, Jiong Yang, Jie Ren, David Voneshen, Shinichiro Asai, Takatsugu Masuda, Suchismita Sarker, Yifei Zhang, Jiangbin Wu, Ping-Heng Tan, Yue Chen, Jie Ma, Yanzhong Pei

**Affiliations:** 1 Interdisciplinary Materials Research Center, School of Materials Science and Engineering, Tongji Univ., 4800 Caoan Rd., Shanghai, China; 2 Department of Mechanical Engineering, The University of Hong Kong, Pokfulam Road, Hong Kong SAR, China; 3 Key Laboratory of Artificial Structures and Quantum Control, School of Physics and Astronomy, Shanghai Jiao Tong Univ., 800 Dongchuan Road, 200240, Shanghai, China; 4 College of Sciences, China Jiliang University, Hangzhou, China; 5 Materials Genome Institute, Shanghai Engineering Research Center for Integrated Circuits and Advanced Display Materials, Shanghai Univ., Shanghai, China; 6 Center for Phononics and Thermal Energy Science, School of Physics Science and Engineering, Tongji Univ., Shanghai, China; 7 ISIS Facility, Rutherford Appleton Laboratory, Didcot, UK; 8 Department of Physics, Royal Holloway University of London, Egham, UK; 9 Institute for Solid State Physics, University of Tokyo, Kashiwanoha, Kashiwa, Chiba, Japan; 10 Institute of Materials Structure Science, High Energy Accelerator Research Organization, Tsukuba, Japan; 11 CHESS, Cornell University, Ithaca, New York, USA; 12 State Key Laboratory of Superlattices and Microstructures, Institute of Semiconductors, Chinese Academy of Sciences, Beijing, China; 13 Center of Materials Science and Optoelectronics Engineering, University of Chinese Academy of Sciences, Beijing, China

**Keywords:** Materials for energy and catalysis, Thermoelectrics

## Abstract

Vortical motion naturally occurs on various scales, from galaxies to skyrmions, influencing the dynamics of mass and information transport. Yet its impact on lattice dynamics in solids is rarely explored so far, primarily owing to the strong interatomic bonds in real materials that inhibit the actual transport of their constituent species. Here we investigate the effect of vortical modes of polarized phonons on the heat insulation of solid materials using synchrotron X-ray diffuse scattering and inelastic neutron scattering along with ab initio molecular dynamics simulations. We demonstrate this vortical vibration as well as its effect on thermal insulation in CsAg_2_I_3_, where the anisotropically bonded iodine atoms form the low-lying transverse optical phonons for enhancing scattering phase space. Moreover, the vortical modes, mainly arising from the chiral symmetric operations acting on the iodine sites, induce two-dimensional short-range-ordering of atomic clusters, lead to a substantially increased phonon scattering centers with a diameter on the order of ~2 primitive cells. This eventually results in extraordinary heat insulation in the single-crystalline CsAg_2_I_3_, exhibiting a thermal conductivity as low as 0.08 W/m-K at room temperature. This strategy establishes a material design paradigm for heat insulation and unveils an unexplored avenue for phonon transport manipulation.

## Abstract (web summary)

The authors reveal that vortical phonon vibrations in CsAg_2_I_3_ induce short‑range ordering, enhancing phonon scattering and yielding an ultralow thermal conductivity of 0.08 W/m‑K, establishing a paradigm for designing heat‑insulation materials.

## Introduction

Within the current fossil fuel-dominated global energy framework, improving thermal energy utilization efficiency has emerged as a critical strategic challenge, given that approximately 60–70% of primary energy is dissipated as waste heat during conversion processes^1^. Thermal insulating materials serve as fundamental materials for suppressing conductive heat transfer, playing a pivotal role in minimizing thermal energy losses during storage and transportation. In addition, it holds significant importance among energy conversion, such as thermoelectric materials^2^. The underlying mechanism revolves around manipulating phononic transport-the dominant heat carriers in crystalline solids-through advanced material design strategies^3–6^.

Heat conduction in crystalline solids dominated by phonon transport is measured by the lattice thermal conductivity (*κ*_L_), which can be quantified by the Boltzmann transport theory via *κ*_L_ = *Cv*^2^*τ*/3. Here, *C* is the lattice specific heat, *v* is the average sound velocity, and *τ* is the phonon relaxation time. Several advanced strategies have been developed and widely employed to manipulate the above three physical parameters^7–9^. Due to the large *v*, acoustic phonons are usually considered as the primary heat carriers in nonmetals. Thus, the transport of long-wave acoustic phonons (aka, mechanical waves) is mostly focused on. Many efforts^10–12^ have concentrated on weakening the interatomic bonds of materials, thus reducing *v* of mechanical waves. However, this would inevitably reduce the mechanical strength of the material as well.

Such a contradiction between heat insulation and mechanical strength can be decoupled by enhancing the scattering strength of short-wave acoustic phonons, as exemplified by the rattler modes observed in thermoelectric clathrates^13^ and skutterudites^14^. In these materials, weakly bonded guest atoms in a cage-like framework induce low-lying optical phonons, leading to a reconstructed acoustic phonon dispersion, an enhanced scattering strength and a low thermal conductivity, while preserving mechanical strength^15^. Inspired by this strategy, similar phonon engineering approaches have been proposed subsequently. For examples, the fast-diffusing Ag ions in argyrodites induce strong phonon scattering between the flat optical modes and acoustic modes^16^; the aliovalent doping in half-Heusler alloys decelerates the acoustic phonons^17^; the twisting modes in higher manganese silicides increase the phonon scattering phase space^18^. As a result, these optical-acoustic phonon coupling mechanisms dramatically suppress the phonon transport.

Although it was analytically proposed that^13^ the low-lying optical phonons can be formed when the second nearest interatomic forces are substantially stronger than the nearest ones, this assumption, however, is somewhat counterintuitive or even unrealistic in most of natural materials. Recent research in phononic crystals^19–21^ indicate that the crystal symmetry of metamaterials can be artificially manipulated to reconstruct the phonon dispersions. By artificially constructing two groups of the *C*_3_ rotational symmetry, vortex modes can be formed, and the modified band-edge establishes acoustic valley states. Alternatively, the simulations suggest that the hydrodynamic phonon transport facilitates the heat dissipation in low-dimensional materials^22–25^.

**Fig. 1:**
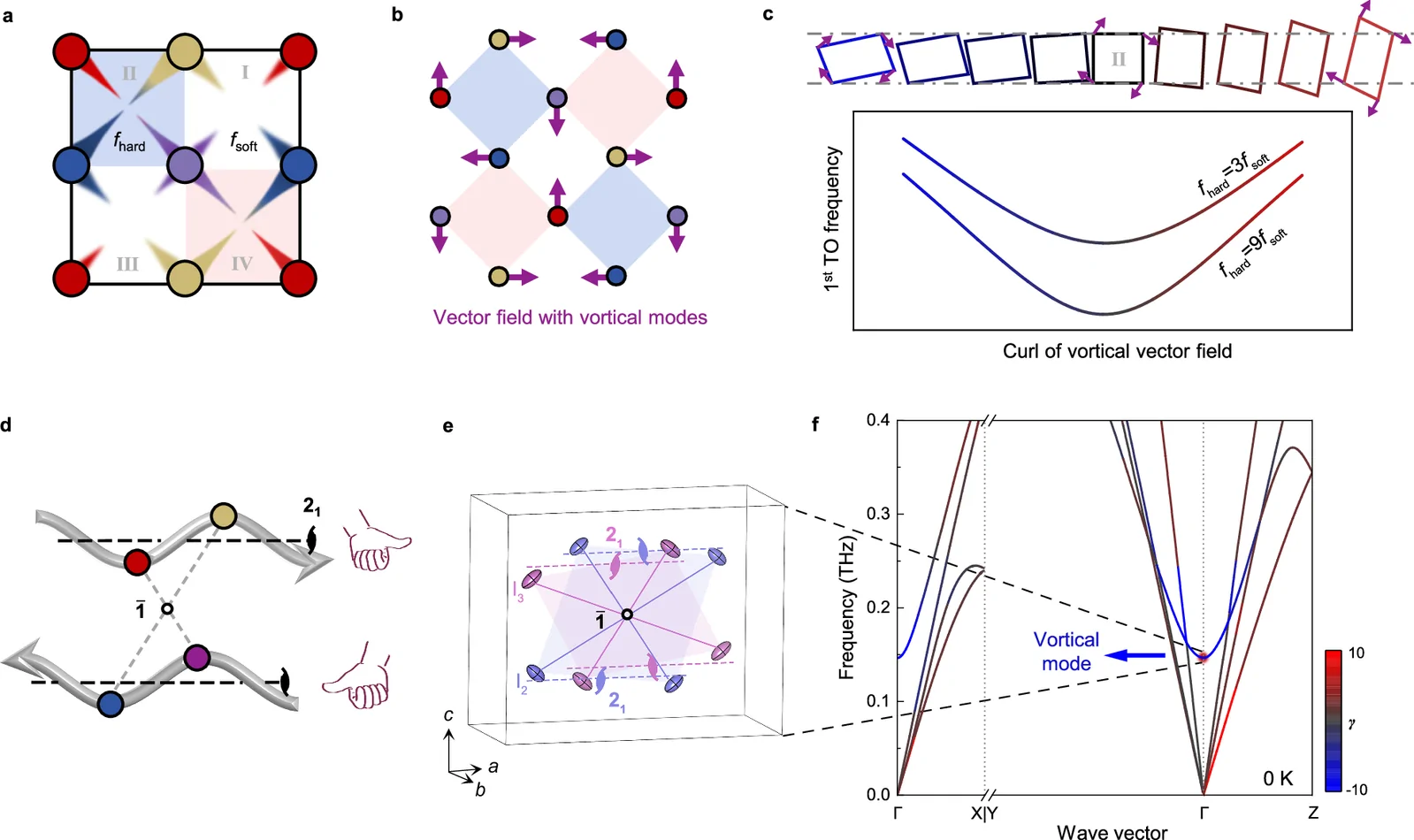
Construction of vortical vibration mode. **a** A two-dimensional square lattice with anisotropic bonds, where the second nearest force constants (*f*_hard_ and *f*_soft_) in II and IV quadrants are stronger than that in I and III quadrants. **b** Eigenvectors of a vortical mode, where the white regions tend to compress while the red/blue regions tend to rotate. The blue and red squares denote the vortical and anti-vortical modes, respectively. **c** Curl dependent 1^st^ transverse optical (TO) frequency for different anisotropic ratios of the second nearest force constants. **d** Symmetric operations of lattice vibration for atomic clusters. **e** Two sets of atomic clusters in CsAg_2_I_3,_ where two Wyckoff 4*c* sites are occupied by iodine atoms (I_2_ and I_3_). The contours of the ellipsoid represent the regions of atomic thermal vibrations. **f** First-principles calculated phonon spectrum at 0 K with the highlight of the characteristic vortical vibration modes. The color bar represents the mode Grüneisen parameter (*γ*).

It is plausible that the construction of specific interatomic bonding and symmetry operations could, in principle, facilitate the formation of low-lying optical modes. Fig. 1a shows a two-dimensional square lattice model illustrating the direction-dependent second nearest interatomic forces, where the second nearest force constants in the II and IV quadrants (*f*_hard_) are stronger than that in the I and III quadrants (*f*_soft_). This anisotropically bonded structure model suggests that the atoms prefer to thermally vibrate along the *f*_soft_ direction, forming a two-dimensional discrete vector field of atomic displacements with vortical and anti-vortical modes (Fig. 1b). In this scenario, the frequency (eigenvalue of phonons) of low-lying optical modes is highly dependent with their eigenvectors, which can be quantified by the curl (aka, local vorticity,*Ω*) of vortical vector field,

1 $${\omega }_{{TO},q=0}\propto {\varOmega }^{2}={\left(\mathop{\sum }\limits_{i}^{n}{{{\bf{v}}}}_{i}{\cdot {{\bf{e}}}}_{i,i+1}/A\right)}^{2}$$

where **v**_*i*_ is the displacement vector of *i*^th^ atom, **e**_*i*,*i*+1_ is the edge vector between the *i*^th^ and *i*+1^th^ atoms, and *A* is the enclosed area of the vector field. As shown in Fig. 1c, for a given bonding strength, the curl can be manipulated by changing the atomic position and the eigenvectors, which substantially affects the frequency of transverse optical (TO) modes (model details in Supplementary Section S1 and Supplementary Fig. S1). When the vortical motions change from elliptical to circular, the TO frequency reaches a minimum. This phenomenon is particularly pronounced in systems with strongly anisotropic chemical bonds.

On this basis, we propose that the low-lying optical modes can be realized in natural crystals via spatial symmetry operations of lattice sites. When a site sequentially undergoes a 2_1_ screw rotation and a $\bar{1}$ inversion, three additional equivalent sites are thus generated (Fig. 1d). The phase differences of the atomic displacements among these four sites sum up to 2π, leading the winding number (aka, global vorticity) to be 1 (mod 2π). If these special sites are occupied by atoms, the related polarized vibrations form vortical modes. Similar to the translational symmetry for long-wave-limit acoustic phonons, it is expected that such vortical modes could induce low-lying optical phonons due to the discrete rotational symmetry, as indicated by Eq. 1 with a certain curl.

This work takes CsAg_2_I_3_ as an example. Very recently, Shen et al.^6^ reported the low thermal conductivity of the polycrystalline CsAg_2_I_3_ at room temperature, which exhibits low sound velocity and strong anharmonicity due to its structural complexity and weak bonding. Here, using synchrotron X‑ray diffuse scattering and molecular dynamics (MD) simulations, we further capture the underlying thermal diffuse scattering arising from vortical modes of polarized phonons in single‑crystalline CsAg_2_I_3_, which is unobserved so far. These results enable a detailed discussion of the structural origin of the anisotropic low thermal conductivity in CsAg_2_I_3_.

The iodine atoms in *P* 2_1_/*n* 2_1_/*m* 2_1_/*a* space group occupy two Wyckoff 4*c* sites (I_2_ and I_3_), both of which allow the above symmetric operations (Fig. 1e). Therefore, two sets of atomic clusters on a sub-unit-cell scale, characterized with anisotropic thermal vibrations, are formed. This is confirmed by first-principles calculations, revealing that a low-lying TO mode originates from vortical vibration with a negative Grüneisen parameter (Fig. 1f). Due to the phase differences in atomic vibrations, the displacement vector field can form dynamic short-range orders with different local vorticity, providing additional scattering centers at the nano scale. Further, due to the increased scattering phase space in the short-wave phonon region, the CsAg_2_I_3_ single crystal exhibits an ultralow thermal conductivity of only 0.08 W/m-K along the *a*-axis at room temperature.

## Results and discussion

The synthesis and phase characterization of both single- and poly-crystalline CsAg_2_I_3_ are given in Supplementary Figs. S2 and S3. Single-crystal X-ray diffraction patterns confirm that CsAg_2_I_3_ has a *Pnma* space group (Supplementary Table S1) and three non-equivalent iodine atom sites are labeled as I_1_, I_2_ and I_3_. Both ab initio molecular dynamics simulations, single-crystal X-ray and neutron powder diffraction structure refinements reveal that the atomic displacements (*U*_11_/*U*_22_/*U*_33_) for Cs, I_2_ and I_3_ are highly anisotropic (Supplementary Table S2 and Supplementary Fig. S4), where the Cs atoms are also part of the overall vortical motion in the lattice and therefore exhibit anisotropic thermal vibrations. The I_1_ and Ag atoms are nearly isotropic, which can be understood by their closer positions to the vortex center. The vortical movements of atomic clusters are thermally activated on a sub-unit-cell scale, where I_2_ and I_3_ atoms move along the normal direction of the (101) plane and Cs along the normal direction of the (100) plane, based on the first-principles calculations (Supplementary Figs. S4d and S5).

**Fig. 2:**
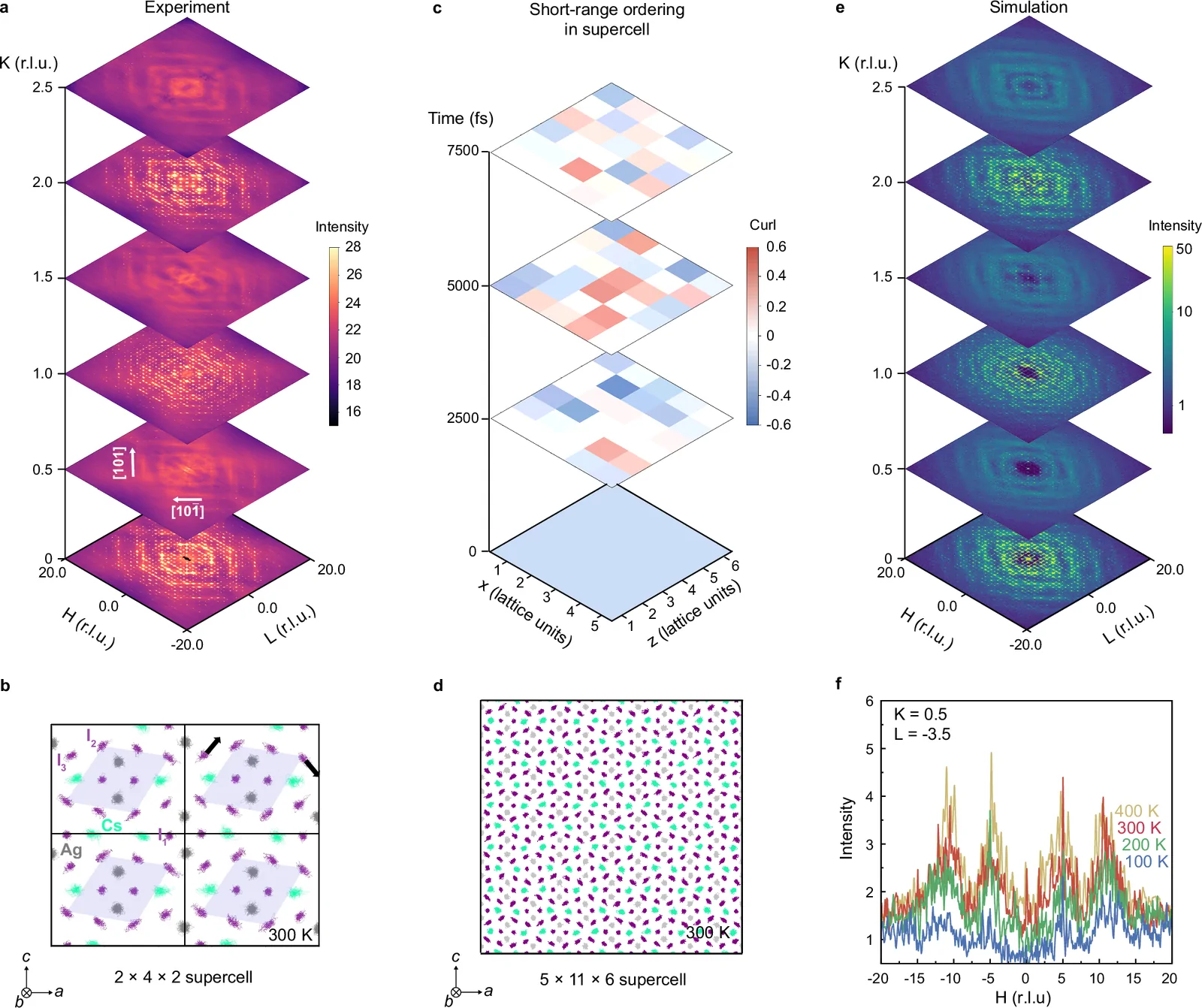
Determination of local vorticity-induced short-range ordering in real and reciprocal spaces for CsAg_2_I_3_. **a** Room-temperature X-ray diffuse scattering of the CsAg_2_I_3_ single crystal at the *Q* = (*HKL*) planes, indicating the existence of short-range ordering. The white arrows mark the direction of the diffuse rods. Room-temperature atomic trajectory projected onto the (010) plane from AIMD in 2 × 4 × 2 supercell (**b**) and from MD in 5 × 11 × 6 supercell (**d**), indicating vortical vibrations, with Cs in green, Ag in gray, and I in purple. **c** Spatiotemporal dynamics of I_2_ rhombi in CsAg_2_I_3_. Each pixel represents the local vorticity at a given time step from the MD atomic trajectory. **e** Theoretical dynamic structure factor (*S*(*Q*)), calculated from MD simulation, at the *Q* = (*HKL*) planes, showing well agreement with measurements. **f** One-dimensional line cut of *S*(*Q*) along *H*, at *K* = 0.5 and *L* = − 3.5.

It is available to experimentally confirm the existence of vortical modes by three-dimensional volumes of X-ray diffuse scattering in reciprocal space. The scattering signals from a single-crystalline CsAg_2_I_3_ were collected at the *Q*-Mapping for Quantum Materials (QM2) beamline at the Cornell High Energy Synchrotron Source (CHESS) using an incident beam energy of 50.0 keV at 300 K^26^. More details of data processing are provided in the Methods section^27^. The three-dimensional volumes of X-ray diffuse scattering data were transformed and represented in reciprocal space coordinates (Fig. 2a; see also Supplementary Fig. S6 for details). For the slices along the b^*^ direction, the Bragg diffraction spots maximize at b^*^ integers and minimize at half-integers. As indicated by the white arrows, the experimental diffuse scattering patterns with the rods along [101] and [$10^{-}1$] directions in reciprocal space can be observed clearly in all the slices. These refer to the directions of the anisotropic displacements for the I_2_ and I_3_ atoms in real space, which is the normal direction of the (101) lattice planes (Fig. 2b, the simulated atomic trajectory based on ab initio molecular dynamics (AIMD) simulation with 384 atoms).

To further investigate the possible short-range order, we performed a large-scale molecular dynamics (MD) simulation with 7920 atoms using the MACE-Omat potential^28,29^, which was optimized for calculating phonon transport behavior. It can be seen that the atomic trajectories from the large-scale MD simulation (Fig. 2d) is highly consistent with those from the small-scale AIMD simulation (Fig. 2b). Given this good agreement, we examine the real-space structure simulated with MD to determine the origin of the one-dimensional (1D) diffuse scattering, which corresponds to two-dimensional (2D) structural correlations in real space.

We show these correlations in Fig. 2c. The plots at various time steps track the average curl (quantified by Eq. 1) for each vortical I_2_ rhombi (as shown in Fig. 2b). The correlated regions of alternating local vortices are identified by neighboring red and blue pixels, which reveal the existence of 2D fluctuating sheets of local vortices with a diameter on the order of ~ 2 primitive cells. The vortical nature of the 1^st^ TO promotes the vortical motion of I_2_ atoms, the vortical regions are confined to a 2D I_2_ rhombi sheet, and the diffuse scattering intensity manifests as 1D rods in the reciprocal space, which is now confirmed by both experiment and the MD simulation results. It is noteworthy that if the local vorticities of all pixels at a given time step are summed, the total local vorticity is zero, indicating the absence of long-range order.

The dynamic scattering factor *S*(*Q*) calculated from the atomic trajectories obtained with MD simulations^30,31^ of CsAg_2_I_3_ is plotted in Fig. 2e. The simulation well reproduces the experimental diffuse scattering, which confirmed the above discussion on the vorticity-induced short-range ordering at the nano scale. It is expected that such nanostructured short-range order could further suppress the thermal conductivity by the fluctuation of phonon frequency, which can be modulated by the local vorticity (Fig. 1c). As illustrated in Fig. 2f, which plots the diffuse scattering intensity against *H* at several temperatures for fixed *K* and *L*, the observed patterns are clearly temperature-sensitive. This behavior points to a phonon-dominated mechanism, confirming that the associated short-range orders are dynamic fluctuations rather than static defect structures (for details on X-ray diffuse scattering from 100 K to 400 K, please refer to Supplementary Fig. S7).

The rocking curves of partial lattice plane derived from single-crystal XRD measurements were carried out to rule out the effect of crystal defects on the diffuse scattering. As shown in Supplementary Fig. S8, the full width at half maximum (FWHM) of rocking curves ranges between 0.5° and 0.6°. Given that the step size of the measurement was 0.5°, the ground-truth FWHM is likely ≤ 0.5°, which is indicative of high crystal quality and rules out significant defect contributions to the observed diffuse scattering.

**Fig. 3:**
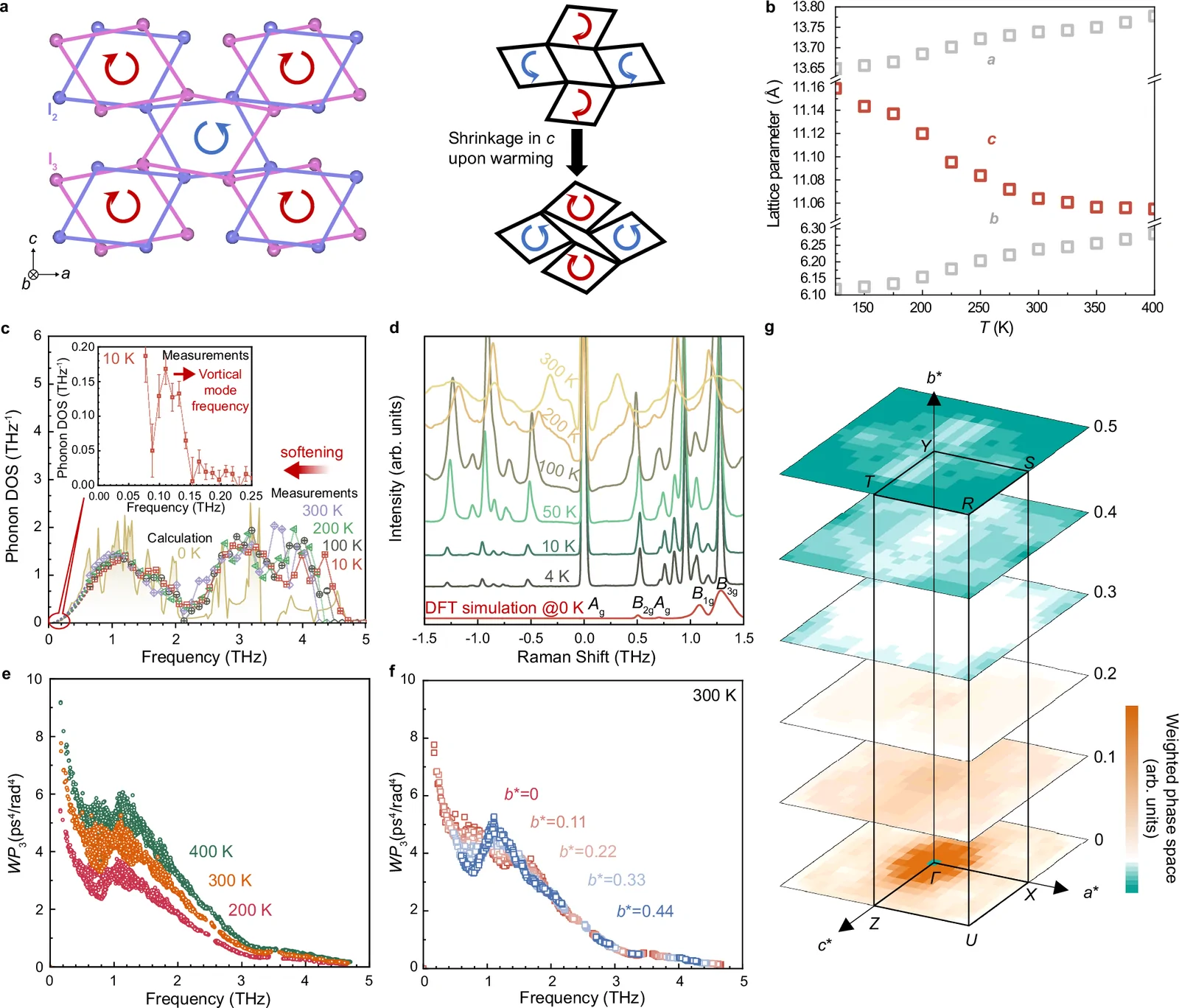
Effects of vortical modes on lattice dynamics for CsAg_2_I_3_. **a** Schematic of negative thermal expansion along the crystallographic *c*-axis induced by vortical modes, along with the experimental evidence of temperature-dependent lattice parameters showing a negative thermal expansion coefficient (**b**). **c** Phonon density of states (DOS) measured by inelastic neutron scattering (dotted lines) and simulated by First-principles calculation (solid line with shaded region) at different temperatures. The error bars represent the statistical uncertainty derived from neutron counting statistics. **d** Temperature-dependent Raman spectra (633 nm excitation), comparing with the DFT-computational Raman-active modes at 0 K. The weighted three-phonon phase space (*WP*_3_) without (**e**) and with (**f**, **g**) momentum resolution. The black box represents the Brillouin zone of space group *Pnma*.

The corner-sharing atom clusters with vortical modes lead to a negative thermal expansion along the *c*-axis direction (Fig. 3a), as the vortical vibrations upon warming shrink the space between polyhedrons. The rotations of vortical and anti-vortical movements at elevated temperatures is very similar to the case of perovskites^30–32^, both lattice structures have rotational sub-lattice units. The large ADPs for Cs, I_2_ and I_3_ indicate the existence of such rotational motions. The macroscopic thermal expansion behavior in CsAg_2_I_3_ is a superposition of positive thermal expansion (dominated by anharmonicity) and negative thermal expansion (dominated by vortical modes). It is speculated that the influence of vortical modes on the *a*-axis is geometrically weaker than on the *c*-axis, because the *a*-axis in CsAg_2_I_3_ is longer than the *c*-axis. This behavior is captured by the temperature-dependent powder X-ray diffraction (Supplementary Fig. S3a), where the crystallographic *c*-axis shows a negative thermal expansion coefficient (Fig. 3b). This is also evident from the negative mode Grüneisen parameter calculated at 0 K (Supplementary Fig. S10c), of which the sudden increase in the absolute value at a very low frequency of 0.15 THz originates from the vortical vibrations, which also indicates the strong anharmonicity^16,33–35^.

First-principles calculations of the phonon spectrum at 0 K reveal an ultralow-frequency TO mode of 0.15 THz, originating from the anisotropic-vibration iodine atoms. This low-frequency optical mode is further verified by the powder inelastic neutron scattering measurements (the inset in Fig. 3c). The measured phonon DOS is overall consistent with the calculated one by first-principles calculations (Supplementary Fig. S9a), and the temperature dependence shows the softening behavior with increasing temperature. We also performed temperature-dependent Raman spectroscopy from 4 to 300 K (Fig. 3d). To exclude potential fluorescence interference, we compared spectra collected using three different excitation wavelengths (Supplementary Fig. S9b). This confirmed that the Raman-active peak represents an intrinsic vibrational signal of the sample, which is supported by the symmetric peak shapes in the Stokes and anti-Stokes lines.

The Raman-active mode of 0.5 THz at 4 K is observed, which softens to 0.3 THz at 300 K. Comparing with the DFT-computational Raman-active modes at 0 K (red line in Fig. 3d, detailed in Supplementary Table S3), we identify that the Raman peak observed corresponds to the second TO mode (*B*_2g_). The DFT simulations at 0 K predict a frequency of 0.5 THz for the 2^nd^ optical mode at the Γ point, which is consistent with the observation by Raman spectra at 4 K. Since the theoretical intensity of the lowest TO mode (*A*_g_) is only about 1/700 of that of the *B*_2g_ mode, it is hard to be detectable in the Raman experiments.

Based on the calculated symmetry characteristics of phonon vibration modes along the Γ-X, Γ-Y, and Γ-Z directions in CsAg_2_I_3_ (Supplementary Fig. S9d–h). It is confirmed that the vortical vibration mode exhibits a crossing characteristic with the acoustic branches, rather than avoided crossing behavior. It is expected that this acoustic-optical branch crossing notably enhances the phonon scattering phase space. For a deeper understanding of the phonon scattering in the presence of vortical modes, we calculate the scattering phase space at elevated temperatures. As illustrated in Fig. 3e and S9c, both the weighted three-phonon (*WP*_3_) and four-phonon (*WP*_4_) scattering phase spaces increase with increasing temperature, indicating increased scattering channels. The difference between *WP*_3_ and *WP*_4_ lies in their frequency dependences, which are dominated by the phonon dispersions.

It is obvious that, taking *WP*_3_ at 300 K as an example, there is a unique characteristic frequency dependence. The *WP*_3_ shows a sudden jump as the frequency reaches the vortical mode region around 0.6 THz, then peaks at 1.2 THz, which is very similar to the flat low-frequency optical modes of YbFe_4_Sb_12_^36,37^. The feature depends on how many momentums of phonons are available for satisfying the energy conservation during scattering processes. Therefore, the *WP*_3_ is highly momentum dependent as shown in Fig. 3f, g.

**Fig. 4:**
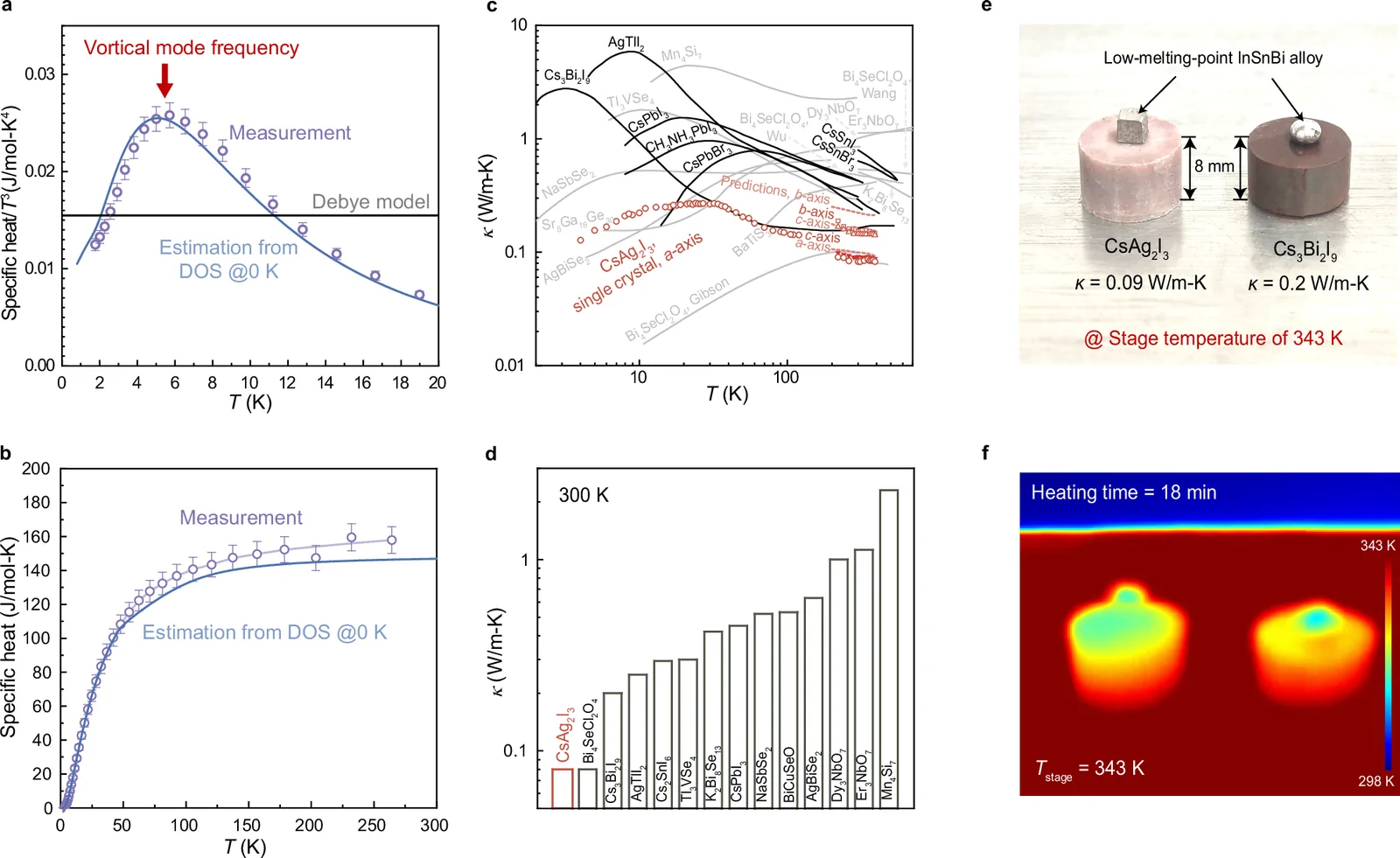
Heat insulation performance for CsAg_2_I_3_. Temperature-dependent specific heat with (**a**) and without (**b**) *T*^3^-normalization, along with the predictions based on the phonon density of states at 0 K and 300 K. The error bars in both panels represent the relative uncertainty of approximately ± 5%, propagated from the sample mass measurement. Temperature-dependent (**c**) and room-temperature (**d**) thermal conductivity (*κ*) compared to that of dense heat insulators^18,41,64–76^, particularly the ternary halides without vortical modes. Comparison of heat insulation between CsAg_2_I_3_ and layered halide materials Cs_3_Bi_2_I_9_^41,42^ with the same-sized cylinders placed on a hot stage of 343 K monitored by optical (**e**) and infrared (**f**) cameras.

The signal of vortical modes would also contribute to the specific heat (for constant pressure, *C*_p_) at low temperatures, the so-called Boson features. According to the Debye model (Eq. S5), the specific heat normalized by the cube of temperature should be independent of temperature (Fig. 4a) at temperatures far below the Debye temperature (91 K for CsAg_2_I_3_, based on Eq. S5). The Boson peak signifies an excess of low-frequency vibrational states beyond the Debye *ω*^2^ prediction. In amorphous materials^38^, this feature is commonly attributed to (quasi-)localized modes induced by structural disorder. However, its presence in crystalline materials^39,40^ indicates a different origin, namely the existence of vibrational modes below the Debye frequency limit. These low-frequency modes provide an additional contribution to the specific heat, leading to a deviation from the Debye *T*^3^ law. The observed Boson peak in CsAg_2_I_3_ at 6 K is in close agreement with the frequency of 0.15 THz of the vortical vibration mode at low temperatures, which can be well reproduced by the calculated phonon DOS at 0 K (Supplementary Fig. S9a). This indicates that the mechanism of the Boson peak is intrinsic due to the additional contribution of the low-energy vortical mode, but not extrinsic due to amorphous-like behavior. On the other hand, the high-temperature specific heat cannot be accurately reproduced from the calculated phonon DOS at 0 K (Fig. 4b), which is primarily attributed to the overall softening of phonons during heating from 0 K to 300 K (Fig. 3c).

As the vortical vibration is deeply wedged into the low-frequency region, the velocity of acoustic phonons (primary heat carriers) is significantly reduced. Both transverse (*v*_t_) and longitudinal (*v*_l_) sound velocities along either the *a*- or *c*-axis, are significantly lower than that along the *b*-axis (Supplementary Fig. S10a) and even lower than that along the cross-plane direction in most two-dimensional halide materials (Supplementary Fig. S10b).

This strong anisotropy in sound velocity can be understood, since the vortical vibrations on a sub-unit-cell scale are confined in the crystallographic *ac* plane (Supplementary Fig. S5a and S5b). Consistently, thermal conductivity (*κ*) is also highly anisotropic, as revealed by *κ* along the *a*-axis being only half of that along the *b*-axis (Fig. 4c). It can be found that CsAg_2_I_3_ exhibits lower thermal conductivity for a wide range of temperatures. More important, such low thermal conductivity in CsAg_2_I_3_ shows a crystal peak at 25 K and does not show glass-like behavior even at 300 K. This indicates that the underlying mechanism for low thermal conductivity in CsAg_2_I_3_ is intrinsic due to the presence of vortical modes and the 2D short-range ordering. Consequently, the temperature-dependent thermal conductivity along *a*-, *b*- and *c*-axes can be well predicted by homogeneous non-equilibrium molecular dynamics (HNEMD) simulations (dashed lines in Fig. 4c and details in Supplementary Fig. S11). Compared to other materials as well as the ternary halides without vortical modes (Fig. 4d), CsAg_2_I_3_ exhibits an ultralow thermal conductivity at 300 K.

The heat insulation behavior of CsAg_2_I_3_ is further confirmed by the infrared camera observations (Fig. 4e, f). The heat insulation performance is compared with that of well-studied crystalline heat insulator Cs_3_Bi_2_I_9_^41,42^, a layered halide material, which is also fabricated in this work. It is noted that: i) the large electronic resistivity > 100 M*Ω*·cm confirms the absence of electronic contribution to thermal conductivity in CsAg_2_I_3_; and ii) the negligible ionic conductivity barely contributes to the reduction of thermal conductivity. Specifically, the additional contribution of silver ion migration to phonon scattering can be ruled out based on two key observations. First, the ionic conductivity, measured by AC impedance spectroscopy, is an intrinsically low level of 10^-7 ^S/cm, which is significantly lower than that of many conventional superionic conductors (Supplementary Fig. S12a). Second, the ion transport channels for Ag atoms in CsAg_2_I_3_ was analyzed by combining the room-temperature X-ray diffraction data and the maximum entropy method (MEM)^43^. It can be seen that the electron density between Ag ions is close to zero, indicating a high electrostatic barrier that impedes Ag ion migration in CsAg_2_I_3_ (Supplementary Fig. S12b).

Both the CsAg_2_I_3_ and Cs_3_Bi_2_I_9_ supports were fabricated to the same height of ~ 8 mm. Since the thermal conductivity for both materials are anisotropic, we performed XRD analysis (Supplementary Fig. S13a) on the cylinders to measure the textures (Supplementary Table S4). It is found that the CsAg_2_I_3_ cylinder exhibits a very weak texture, while the Cs_3_Bi_2_I_9_ one has a pronounced preferential orientation along the *c*-axis. The thermal conductivity of Cs_3_Bi_2_I_9_^42^ along the *c*-axis is higher than that perpendicular to the *c*-axis. Noted that, the thermal conductivity along the principal axes in Cs_3_Bi_2_I_9_ exceeds that in CsAg_2_I_3_ (Supplementary Fig. S13b). As shown in Fig. 4e, f and Supplementary Fig. S14, both materials of the same size are placed on a heat stage of 343 K. The melting of an InSnBi alloy (melting point of 320 K) sitting on top of Cs_3_Bi_2_I_9_ suggests its inferior thermal insulation to that of CsAg_2_I_3_.

In summary, the effects of anisotropic bonding on vortical modes and heat insulation of CsAg_2_I_3_ are comprehensively investigated. The anisotropic thermal vibrations of iodine atoms introduce low-lying optical modes. The collective vibrations on a sub-unit-cell scale arising from the chiral symmetric operations acting on the lattice sites of iodine atoms enable the vortical modes to dramatically reduce the thermal conductivity in CsAg_2_I_3_. This mechanism leads to the realization of an extraordinarily low thermal conductivity of 0.08 W/m-K near room temperature in single crystalline CsAg_2_I_3_, suggesting potential for advancing heat insulation and thermoelectricity. The underlying physics, governed by discrete rotational symmetry arising from specific Wyckoff sites, suggests a potentially universal design principle for ultralow thermal conductivity in anisotropic crystals. Given that the key symmetric operations exist in other space groups (*e.g*., Wyckoff 8 *g* sites in *C* 2/*m* 2/*c* 2_1_/*m*), it is reasonable to expect similar vortical phonon modes and their pronounced scattering effects in a broader family of structurally anisotropic materials.

## Methods

### Synthesis

High-purity CsI (99.9%, Aladdin) and AgI (99.99%, Aladdin) mixtures with stoichiometric proportions were loaded and sealed in the quartz ampoules under vacuum. For the synthesis of poly-crystalline CsAg_2_I_3_, the ampoule was heated up to 1073 K and held for 2 h, then cooled to 623 K and quenched in cold water, followed by annealing at 423 K for 48 h. The resulting ingot was hand ground into fine powder for hot pressing at 393 K for 10 minutes. The dense pellets were obtained (> 97% of the theoretical density) and further fabricated into various geometries for different measurements. A vertical temperature gradient freeze technique was used for the growth of single-crystalline CsAg_2_I_3_. As the pre-synthesis, the ampoule was heated up to 1073 K for 2 hours for pre-melting, and then cooled down to room temperature. This ampoule was then placed in a vertical temperature gradient furnace. The tip position of the ampoule was heated to 483 K holding for 2 h, and then slowly cooled down at a rate of ~ 0.7 K/h (Supplementary Fig. S2).

Cs_3_Bi_2_I_9_ for infrared cameras was synthesized by sealing stoichiometric mixtures of high-purity CsI (99.9%, Aladdin) and BiI_3_ (98%, Aladdin) in quartz ampoules under vacuum. The ampoule containing the above powder mixture was heated up to 973 K and held for 2 h, then quenched in cold water. Subsequently, the ingot continued to anneal at 573 K for 48 h. The annealed ingot then was hand ground into fine powder for hot pressing at 550 K for 20 min. Thus, a dense bulk (> 97% of the theoretical density) with ~ 12 mm in diameter and ~ 8 mm in thickness was obtained.

### Structural and compositional characterization

Temperature-dependent X-ray diffraction (XRD, Bruker D8 Advance, Cu-K*α* radiation) of CsAg_2_I_3_ powder shows no phase transition or phase decomposition up to 400 K (Supplementary Fig. S3a). The derived temperature-dependent lattice parameters have uncertainties on the order of 0.1‰, stemming predominantly from the refinement of the XRD patterns. The crystal orientations of both the single crystal (Supplementary Fig. S3b) and the polycrystalline CsAg_2_I_3_ and Cs_3_Bi_2_I_9_ cylinders (Supplementary Fig. S13a) characterized by X-ray diffraction (XRD, Rigaku SmartLab, Cu-K*α* radiation). The diffraction data of single crystals were also collected by a single crystal X-ray diffractometer (Bruker D8 VENTURE diffractometer with a PHOTON II CCD detector, Mo-K*α* radiation). The single crystal structure was then resolved and refined by SHELXT and OLEX2^44,45^. The detailed crystallographic parameters are listed in Supplementary Table S1. The composition of single crystalline CsAg_2_I_3_ was characterized by Scanning Electronic Microscopy (SEM, Phenom Pro) equipped with an Energy Dispersive Spectrometer (EDS), confirming the homogenous composition (Supplementary Fig. S3c). A typical photograph of the single crystalline CsAg_2_I_3_ bulk was showed in Supplementary Fig. S3d. The neutron powder diffraction experiment was carried out at the multi-physics instrument (MPI) at China Spallation Neutron Source (CSNS)^46^. Polycrystalline CsAg_2_I_3_ powders with mass 3.51 g were loaded into a 9 mm inner-diameter Vanadium Nickel can with a sample height of 28 mm. The Rietveld refinement was performed against the diffraction data from Bank 7, which has high reciprocal-space resolution and a wide *Q* range, using the GSAS-II software^47^. The detailed crystallographic parameters are listed in Supplementary Table S2.

### Three-dimensional synchrotron X-ray diffuse scattering

Three-dimensional synchrotron X-ray diffuse scattering measurements were carried out at the Quantum Materials (QM2) beamline at the Cornell High Energy Synchrotron Source (CHESS)^26^. The CsAg_2_I_3_ single crystal was measured at 300 K using an incident beam energy of 50.0 keV. A PILATUS 6 M Silicon two-dimensional detector was mounted behind the sample perpendicular to the primary beam path with a sample-to-detector distance of 400.63 mm. During the measurements, the samples were continuously rotated about an axis perpendicular to the beam at 1° per second over 365°, with images read out every 0.1 s. Three sets of rotation images at *χ* angles (90°, 85°, 95°) were collected for the CsAg_2_I_3_ sample to fill in detector gaps and allow for the removal of detector artifacts. The resulting images were stacked into a three-dimensional array and transformed into reciprocal space coordinates using the software packages NXRefine and CCTW (Crystal Coordinate Transformation Workflow) within NeXpy^48^. The resulting structure function *S*(*Q*) was determined over a range of ± 27 Å^−1^, ± 16 Å^−1^, and ± 29 Å^−1^ in the *H*, *K*, and *L* reciprocal lattice directions, respectively.

### Inelastic neutron scattering

The inelastic neutron scattering (INS) experiments were conducted on the High Resolution Chopper Spectrometer, HRC, at the Materials and Life Science Experimental Facility (MLF), the Japan Proton Accelerator Research Complex (J-PARC), and the time-of-flight spectrometer MERLIN, ISIS Neutron and Muon Source of UK. Approximately 10.3 grams samples were ground into powder and loaded into a thin-walled aluminum can. The INS sample data and empty can data were collected with a multi-incident energy mode of *E*_*i*_s as 40.66 meV and 10.25 meV over the temperature range of ~ 10 K to 300 K with a closed-cycle refrigerator. The dynamic structure factors, *S*(*Q*, E), were processed by DAVE software^49^, and we further extracted neutron-weighted phonon DOSs by integrating the *S*(*Q*, E) over the range of moment transfers from 0.5 to 4.5 Å using the GetDOS program^50^, and the energy resolution is around 5%.

### Thermal transport measurements

For temperature region from 220 K to 390 K, the thermal conductivity (*κ*) is measured by the laser flash method (Netzsch LFA467) via *κ* = *ρC*_p_*λ*, where *ρ* is the density, *C*_p_ is the specific heat (using the Dulong-Petit limit of specific heat in this work), *λ* is the thermal diffusivity. The errors of ± 2–4% stem from the multiple measurements at each temperature. All samples were 0.7–1 mm thick and tested in an argon atmosphere. For temperature region from 4 K to 120 K, the thermal conductivity was measured by the steady-state method (Multifield Technology Co.) via *κ* = *PL*/(Δ*TS*), where *P* is the heater power, *L* is the length between two thermocouple contacts, Δ*T* is the temperature difference after stabilization of heating and before heating and *S* is the cross-sectional area of the sample, and the errors of ±2% are primarily subject to dimensional measurement error between thermocouples. All measurements were taken under a high vacuum < 10^−5 ^Pa. The sample used in the test was about 1.5 × 3.5 × 4 mm^3^ in size. The error bars have been omitted for temperature-dependent thermal conductivity, since the size of the error bars are all smaller than the size of the symbols.

### Specific heat and sound velocity

The specific heat was measured in a physical properties measurement system (PPMS, Quantum Design) for the temperature range from 2 K to 300 K, with the primary source of error from the mass measurement of the sample, which introduces an error of around ± 5%. The sound velocity was measured at room temperature using an ultrasonic pulse receiver (Olympus-NDT) equipped with an oscilloscope (Keysight), which is accompanied by an error of approximately ± 5% attributed to the measurement deviation in the sample’s thickness.

### Raman spectroscopy

Raman spectra are measured in back-scattering at room temperature using a Jobin-Yvon HR800 micro- Raman system equipped with a liquid-nitrogen-cooled charge couple detector (CCD). For low-temperature measurements, a closed-cycle cryostat system (attoDRY800, Attocube Systems AG) was used to cool the sample to 4 K. In this cryogenic configuration, a high-NA objective (NA = 0.82) was employed to optimize the signal collection efficiency. The excitation wavelengths are 488 nm, 532 nm and 633 nm from an Ar^+^ laser, a diode-pumped solid-state laser and a HeNe laser, respectively. An 1800 lines/mm grating enables us to have each pixel of the charge-coupled detector cover ~ 0.35 cm^-1^ at 633 nm. The Raman system is equipped with a 10 × objective lens (numerical aperture (NA) of 0.25). The laser plasma lines are removed by BragGrate bandpass filters (OptiGrate Corp.), since these lines would appear in the same spectral range as the low-frequency Raman modes. Measurements down to 5 cm^-1^ are achieved by three BragGrate notch filters (OptiGrate Corp.) to suppress the Rayleigh line with an optical density of 4 and with a full width at half maximum of 5 ~ 10 cm^-1^ ^51^. The laser power is kept below 0.4 mW to avoid sample heating.

### First-principles calculations and lattice dynamics

Vienna *Ab*-*initio* Simulation Package (VASP)^52^ based on the projector augmented wave (PAW)^53^ method was employed to perform first-principles calculations. The Perdew–Burke–Ernzerhof for solids (PBEsol) functional^54^ within the generalized gradient approximation (GGA)^55^ was used to calculate the exchange-correlation energy. A plane-wave energy cutoff of 520 eV and a sampling spacing of about 2π × 0.02 Å^−1^ with Γ-centered Monkhorst-Pack scheme in the Brillouin zone were adopted for structural relaxations. The convergence criteria of atomic force and energy were set to 10^−3 ^eV/Å and 10^−7 ^eV, respectively.

For calculating the phonon property, a 2 × 4 × 2 supercell (384 atoms) of CsAg_2_I_3_ was used. The mode Grüneisen parameters were calculated based on the finite difference method as implemented in the phonopy package^56^ with a volume variation of ± 0.3%. The finite-displacement method with a displacement of 0.01 Å was used to calculate the phonon dispersions at 0 K. To extract the renormalized IFCs, we carried out ab initio molecular dynamics (AIMD) simulations at 300 K with an energy cutoff of 350 eV and a Γ-centering 1 × 1 × 1 *k*-point mesh. The AIMD time step was set to 2.0 fs and 5500 steps were conducted. 50 configurations were randomly extracted from the AIMD trajectories, and we raised the energy cutoff to 520 eV with a 1 × 1 × 1 *k*-point mesh to perform accurate static calculations to obtain the atomic forces. Atomic anisotropic displacement parameters were also computed from AIMD trajectories. The neighbor cutoff distances of 8.0, 6.0 Å and 5.0 Å were chosen to fit the second-, third- and fourth- order IFCs of CsAg_2_I_3_, respectively. The atomic trajectory was extracted from a long AIMD simulation with 6950 steps. The weight three-/four-phonon scattering phase space were calculated using the ShengBTE package^57^ with a *q*-grid of 17 × 10 × 21 and 4 × 9 × 5, respectively.

### Molecular dynamics simulations

For the analysis of short-range order, molecular dynamics (MD) simulations were performed using a 5 × 11 × 6 supercell containing 7920 atoms. The simulations utilized the MACE-Omat potential^28,29^. The system was first equilibrated in the NVT ensemble for 1.25 ps, followed by a production run in the NVE ensemble for 7.25 ps. The atomic velocities were collected at an interval of 10.0 fs during the final 6.0 ps of the trajectory for subsequent analysis. All MD simulations were carried out using the Large-scale Atomic/Molecular Massively Parallel Simulator (LAMMPS) package^58^ with a time step of 0.25 fs. The dynamical structure factor, *S*(*Q*) are generated using identical simulations to those in Sterling and Toney^30,59^.

### Lattice thermal conductivity calculations by HNEMD

We used the homogeneous nonequilibrium molecular dynamics method (HNEMD)^60,61^ as implemented in the Graphics Processing Units Molecular Dynamics (GPUMD) package^62^ to compute the lattice thermal conductivity of CsAg_2_I_3_. To minimize the size effect on the heat flux calculation, we adopted an 11 × 26 × 13 supercell containing 89232 atoms after careful testing (details in Supplementary Supplementary Fig. S11). The simulation system was first equilibrated under the NVT ensemble for 500 ps, followed by a 2.0 ns HNEMD simulation in the same ensemble to extract the heat flux. The target temperatures (200, 250, 300, 350, and 400 K) were controlled by a Nosé-Hoover chain thermostat^63^ with a thermostat coupling constant of 0.1 ps. The data were averaged every 1000 steps before recording, and the driving force parameter was set to 2 × 10^-4 ^Å^-1^. 15 independent HNEMD simulations were performed for the heat flux in CsAg_2_I_3_ at each temperature to obtain the average directional lattice thermal conductivity and its statistical error.

## Supplementary information

Supplementary Information

Transparent Peer Review file

## Data Availability

The authors declare that all data supporting the findings of this study are available within the article and its Supplementary Information files.

## Code availability

The open-source codes are available as follows: Vienna Ab-initio Simulation Package (VASP) is available at https://www.vasp.at/. MACE-Omat potential is available at https://github.com/ACEsuit/mace-foundations. The LAMMPS package is available at https://www.lammps.org/. ShengBTE package is available at https://www.shengbte.org/. Pynamic-structure-factor is available at https://github.com/tyst3273/pynamic-structure-factor. The Graphics Processing Units Molecular Dynamics (GPUMD) package is available at https://github.com/brucefan1983/GPUMD. The in-house codes used in this work are available from the corresponding authors upon reasonable request.
