# Supplementary material for: Vorticity-induced short-range ordering for heat insulation in anisotropically bonded CsAg2I3
Source: Nat Commun. 2026 Aug 21;17:10344. doi: 10.1038/s41467-026-76794-9 (PMC13624264; doi:10.1038/s41467-026-76794-9)
Supplement: Supplementary file 1 [file 41467_2026_76794_MOESM1_ESM.pdf]

Section S1: Phonon dispersion modeling

For a square lattice as shown in **Figure S1**, the primitive cell contains 4 atoms (atoms 0, 1, 2 and 5). Since each atom has 2 in-plane degrees of freedom, the dynamical matrix is a 8×8 Hermitian matrix[1],

$$D(\boldsymbol{q}) = \begin{pmatrix} D_{xx}^{00} & D_{xy}^{00} & D_{xx}^{01} & D_{xy}^{01} & D_{xx}^{02} & D_{xy}^{02} & D_{xx}^{05} & D_{xy}^{05} \\ D_{yx}^{00} & D_{yy}^{00} & D_{yx}^{01} & D_{yy}^{01} & D_{yx}^{02} & D_{yy}^{02} & D_{yx}^{05} & D_{yy}^{05} \\ D_{xx}^{10} & D_{xy}^{10} & D_{xx}^{11} & D_{xy}^{11} & D_{xx}^{12} & D_{xy}^{12} & D_{xx}^{15} & D_{xy}^{15} \\ D_{yx}^{10} & D_{yy}^{10} & D_{yx}^{11} & D_{yy}^{11} & D_{yx}^{12} & D_{yy}^{12} & D_{yx}^{15} & D_{yy}^{15} \\ D_{xx}^{20} & D_{xy}^{20} & D_{xx}^{21} & D_{xy}^{21} & D_{xx}^{22} & D_{xy}^{22} & D_{xx}^{25} & D_{xy}^{25} \\ D_{yx}^{20} & D_{yy}^{20} & D_{yx}^{21} & D_{yy}^{21} & D_{yx}^{22} & D_{yy}^{22} & D_{yx}^{25} & D_{yy}^{25} \\ D_{xx}^{50} & D_{xy}^{50} & D_{xx}^{51} & D_{xy}^{51} & D_{xx}^{52} & D_{xy}^{52} & D_{xx}^{55} & D_{xy}^{55} \\ D_{yx}^{50} & D_{yy}^{50} & D_{yx}^{51} & D_{yy}^{51} & D_{yx}^{52} & D_{yy}^{52} & D_{yx}^{55} & D_{yy}^{55} \end{pmatrix} \qquad \text{S1}$$

The matrix element is

$$D_{xy}^{ij}(\boldsymbol{q}) = (M_i M_j)^{-1/2} \sum_{l} \phi_{xy} \begin{pmatrix} i & j \\ l & l` \end{pmatrix} e^{-\mathrm{i}\boldsymbol{q}\cdot\boldsymbol{R}_{ij}} \qquad \text{S2}$$

where M_i and M_j are the masses of the ith and j^{th} atoms, x and y represent components of Cartesian coordinate axes, $\phi^{ij}(i,j;l,l`)$ is the force acting on the $i^{th}$ atom in the $l^{th}$ primitive cell along $x$ direction due to the displacement of the $j^{th}$ atom in the $l`^{th}$ primitive cell along y direction, $\boldsymbol{q}$ is the wave vector of phonons, $\boldsymbol{R}_{ij}$ is the difference of lattice translation vectors between i^{th} and j^{th} atoms. $\phi^{ij}(i,j;l,l`)$ can be expressed as,

$$\phi_{xy} \begin{pmatrix} i & j \\ l & l` \end{pmatrix} = -f_{ij}\boldsymbol{r}_{ij,x} \cdot \boldsymbol{r}_{ij,y} \qquad \text{S3}$$

where $\boldsymbol{r}_{ij}$ is the unit vector along $\boldsymbol{R}_{ij}$, f_{ij} is the second-order force constant between i^{th} and j^{th} atoms. In this work, we only consider the nearest (f_n, e.g., f_{01}), the second nearest (f_{nn}, e.g., f_{05}) and the third nearest (f_{nnn}, e.g., f_{09}) neighbor force constants. Hence, the involved atoms in $\phi^{ij}(i,j;l,l`)$ are shown in **Figure S1a**.

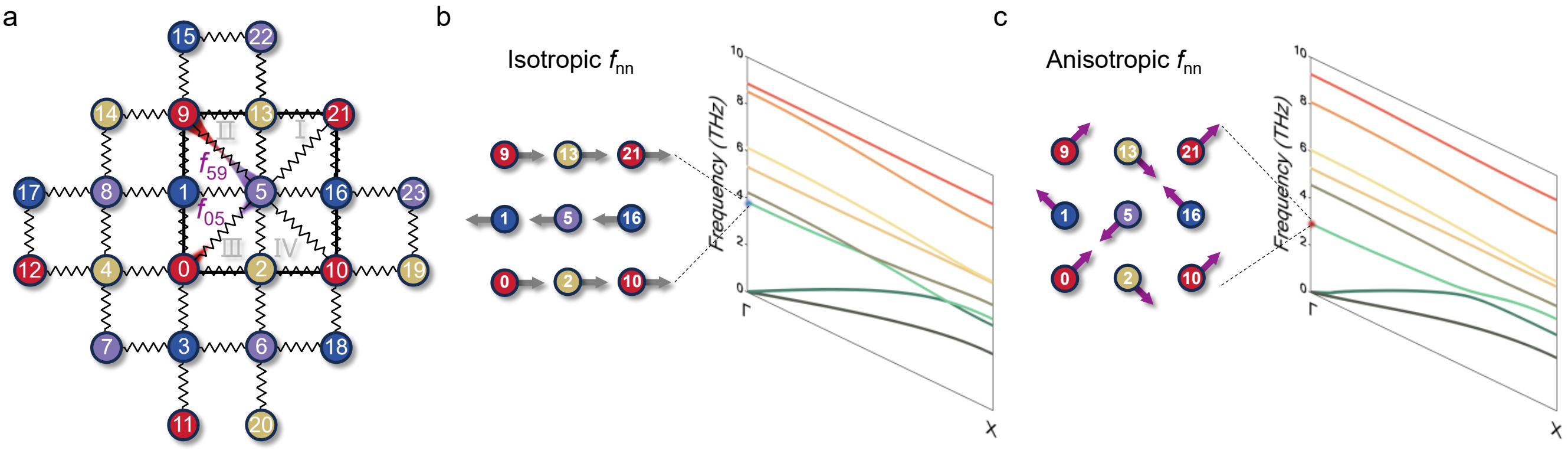

Figure S1. Lattice dynamics model and calculated phonon dispersions. (a) A two-dimensional square lattice for lattice dynamic model. The black box represents the primitive cell, which contains four atoms. The springs describe the nearest and the second nearest forces between atoms labeled by numbers. The phonon spectra for the cases of isotropic (b) and anisotropic (c) second nearest force constants, where the arrows represent the eigenvectors of the 1st optical branch at Γ point.

The phonon dispersions and vibrational modes can be obtained by solving the eigenvalues and eigenvectors of the dynamical matrix. After some numerical simulations, we found that f_{nnn} are unimportant and can be ignored in this model. It is also assumed that all the f_n are identical for simplicity. That is, the effect of f_{nn} on the phonon dispersion is primarily focused on. In the following numerical simulations, the force constants and masses are set as follow: $f_{nnn} = 0$, $f_n = 20$, $M_0 = 1$, $M_1 = 2$, $M_2 = 3$, $M_5 = 4$. To keep the average second nearest forces unchanged, $f_{nn} = 10$ for the isotropic case (**Figure S1b**) while $f_{05} = f_{12} = f_{521} = f_{1316} = 5$ and $f_{113} = f_{59} = f_{216} = f_{510} = 15$ for the anisotropic case (**Figure S1c**). At $q = 0$, it can be found that the frequency of the 1st optical modes in the anisotropic case is much lower than that in the isotropic case. More important, the eigenvectors are the horizontal motions in the isotropic case but are vortical motions in the anisotropic case. This can be understood by the certain eigenvectors with a large component modulating the soft second nearest forces.

The eigenvectors of a certain phonon mode constitute a two-dimensional discrete vector field, of which the rotational characteristics can be described by the average curl. For an eigenvector $\mathbf{v}_i = (\boldsymbol{v}_{i,x}, \boldsymbol{v}_{i,y})$ defined at lattice position of ith atom, the local vorticity (curl, Ω) can be approximated by the circulation (Γ) around a small closed contour divided by the enclosed area (A),

$$\Omega = \frac{\Gamma}{A} = \frac{\sum_i^n \mathbf{v}_i \cdot \mathbf{e}_{i,i+1}}{A} \qquad \text{S4}$$

where $\mathbf{e}_{i,i+1}$ is the edge vector between the ith and $i+1^{th}$ atoms. Ω represents the local rotation strength of the vector field, analogous to the rotation of a fluid element in a flow. An increase or decrease in curl means that the rotation of the vector field is stronger or weaker. Equation S4 suggests that the curl can be manipulated by changing the position of atoms. To investigate the effect of curl on the low-lying optical modes, the positions of the red and purple atoms are set to be closer to or farther away from each other by

an equal distance to maintain a constant area of the closed loop. It is concluded that the frequency of the low-lying optical mode reaches the minimum when the vortical motions change from elliptical to circular. This phenomenon is particularly pronounced in systems with strongly anisotropic chemical bonds.

Section S2: Synthesis and methods

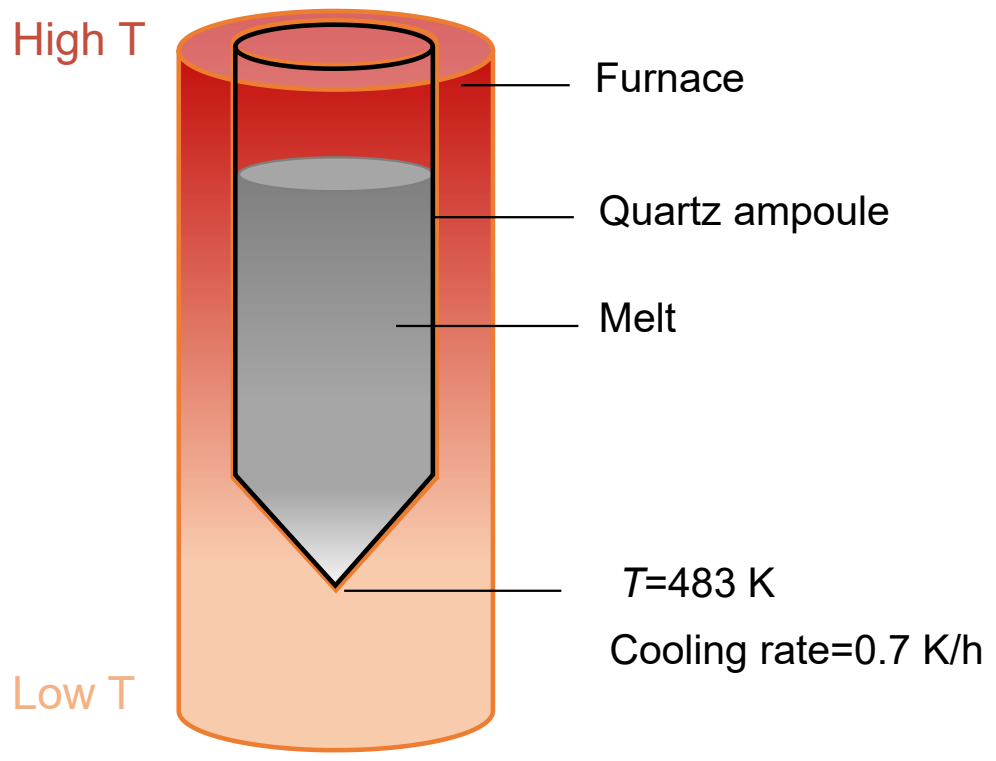

Figure S2. Schematic of the growth of $CsAg_2I_3$. The figure illustrates the vertical temperature gradient freeze technique employed for crystal growth.

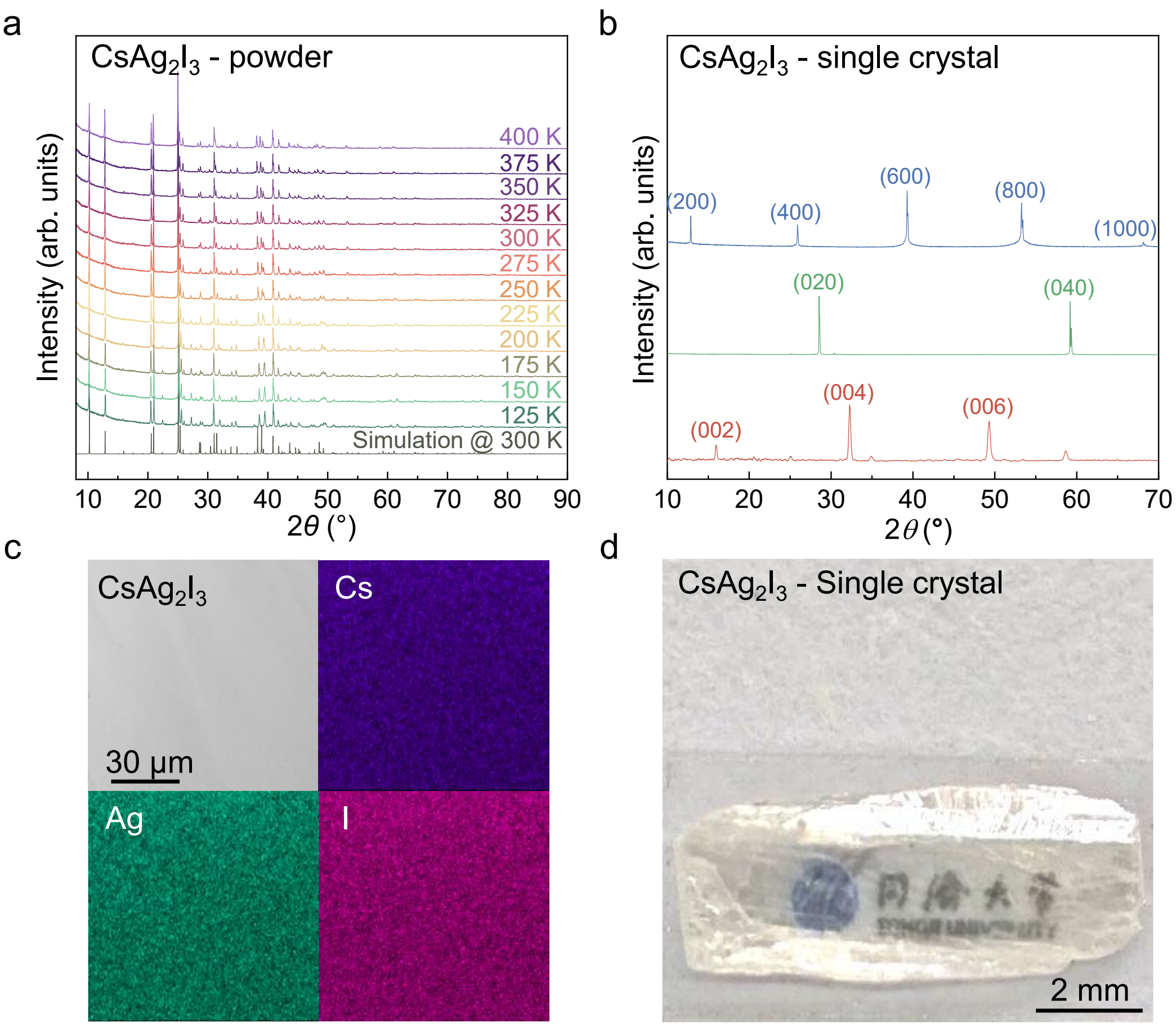

Figure S3. Characterization of $CsAg_2I_3$ crystal structure and morphology. (a) Temperature-dependent powder X-ray diffraction patterns for $CsAg_2I_3$. (b) X-ray diffraction patterns to the (100)/(010)/(001) planes. (c) SEM and EDS images of the cleaved surfaces. (d) A typical photograph of the single crystalline $CsAg_2I_3$.

Table S1. Crystallographic data from single-crystal X-ray diffraction refinements at 300 K.

Formula	$CsAg_2I_3$
M_r, g·mol^{-1}	729.3553
Crystal size, mm^3	0.4×0.4×0.3
Crystal system	orthorhombic
Space group	*Pnma*
T, K	300
a, Å	13.7456(13)
b, Å	6.2327(5)
c, Å	11.0843(10)
α, °	90
β, °	90
γ, °	90
V, Å^3	949.62(15)
Z	4
D_{calcd}, g·cm^{-3}	5.1129(28)
F(000)	1232
μ, mm^{-1}	17.561
θ range, °	3.68-31.94
reflections collected	5259
independent reflections (R_{int})	1687(0.0356)
reflections observed [$I > 2\sigma(I)$]	1222
data/restraints/parameters	1687/0/35
R_1, wR_2 ($I > 2\sigma(I)$)	0.0841, 0.2062
R_1, wR_2 (all data)	0.1096, 0.2365
GooF on F^2	1.118
$\Delta\rho_{max}$, $\Delta\rho_{min}$, e·Å^{-3}	3.979/-4.832
position $x/y/z$ of Cs	0.31643(13)/0.250000/0.01066(12)
position $x/y/z$ of Ag	0.49847(6)/0.49953(11)/0.33754(10)
position $x/y/z$ of I_1	0.62336(7)/0.750000/0.49663(7)
position $x/y/z$ of I_2	0.35997(11)/0.750000/0.21683(13)
position $x/y/z$ of I_3	0.62145(12)/0.250000/0.19440(14)
mean-square displacements $U_{11}/U_{22}/U_{33}/U_{12}/U_{13}/U_{23}$ of Cs (Å^2)	0.1017(12)/0.0384(6)/0.0822(10)/0/-0.0096(6)/0
mean-square displacements $U_{11}/U_{22}/U_{33}/U_{12}/U_{13}/U_{23}$ of Ag (Å^2)	0.0600(6)/0.0491(6)/0.0677(7)/0.0080(4)/-0.0003(3)/-0.0022(3)
mean-square displacements $U_{11}/U_{22}/U_{33}/U_{12}/U_{13}/U_{23}$ of I_1 (Å^2)	0.0322(5)/0.0443(6)/0.0451(6)/0/0.0016(3)/0
mean-square displacements $U_{11}/U_{22}/U_{33}/U_{12}/U_{13}/U_{23}$ of I_2 (Å^2)	0.0808(9)/0.0418(6)/0.0713(8)/0/-0.0396(6)/0
mean-square displacements $U_{11}/U_{22}/U_{33}/U_{12}/U_{13}/U_{23}$ of I_3 (Å^2)	0.0950(10)/0.0437(6)/0.0802(9)/0/0.0511(7)/0

Table S2. Crystallographic data from neutron powder diffraction Rietveld refinement at 300 K.

Formula	$CsAg_2I_3$
Crystal system	orthorhombic
Space group	*Pnma*
T, K	300
a, Å	13.7381(5)
b, Å	6.22243(13)
c, Å	11.0776(4)
α, °	90
β, °	90
γ, °	90
V, $Å^3$	946.96(3)
Z	4
R_w	0.02202
position $x/y/z$ of Cs	0.3160(5)/0.250000/0.0115(10)
position $x/y/z$ of Ag	0.5004(6)/0.5025(11)/0.3375(4)
position $x/y/z$ of I_1	0.6244(4)/0.750000/0.4973(8)
position $x/y/z$ of I_2	0.3630(10)/0.750000/0.2180(7)
position $x/y/z$ of I_3	0.6231(7)/0.250000/0.1883(8)
mean-square displacements $U_{11}/U_{22}/U_{33}/U_{12}/U_{13}/U_{23}$ of Cs ($Å^2$)	0.106(5)/0.0384(6)/0.0822(10)/0/-0.0096(6)/0
mean-square displacements $U_{11}/U_{22}/U_{33}/U_{12}/U_{13}/U_{23}$ of Ag ($Å^2$)	0.0556(26)/0.0375(16)/0.0596(23)/0.016(4)/0.033(7)/-0.030(5)
mean-square displacements $U_{11}/U_{22}/U_{33}/U_{12}/U_{13}/U_{23}$ of I_1 ($Å^2$)	0.036(4)/0.0332(26)/0.042(3)/0/0.010(11)/0
mean-square displacements $U_{11}/U_{22}/U_{33}/U_{12}/U_{13}/U_{23}$ of I_2 ($Å^2$)	0.137(9)/0.029(4)/0.049(5)/0/-0.046(11)/0
mean-square displacements $U_{11}/U_{22}/U_{33}/U_{12}/U_{13}/U_{23}$ of I_3 ($Å^2$)	0.100(8)/0.008(3)/0.105(7)/0/0.103(13)/0

According to the Debye approximated phonon density of states (DOS), the cube of temperature (T^3) normalized specific heat at constant volume (C_v) can be written as Equation S5 when the temperature is far below the Debye temperature (Θ~91 K for $CsAg_2I_3$),

$$C_V = \frac{12\pi^4}{5} NR(\frac{T}{\Theta})^3, (T \to 0 \text{ K}) \qquad \text{S5a}$$

where the N is the number of atoms in chemical formula (N=6 for $CsAg_2I_3$) and the R is the gas constant. The more generalized expression for C_v is given in Equation S5b,

$$C_V = NR \int \frac{(\frac{\hbar\nu}{k_B T})^2 e^{\hbar\nu/k_B T}}{(e^{\hbar\nu/k_B T} - 1)^2} \text{DOS} d\nu \qquad \text{S5b}$$

where $\hbar$ is the reduced Plank constant, ν is the phonon frequency, k_B is the Boltzmann constant.

Notably, according to the Debye model, the Debye temperature (θ_D) is calculated by the following equation,

$$\theta_D = \frac{\hbar}{k_B}(\frac{6\pi^2}{V})^{\frac{1}{3}} v_D \qquad \text{S6}$$

where V is the average atomic volume, v_D is the average sound velocity estimated from transverse (v_t) and longitudinal (v_l) sound velocities via $1/v_D^3 = (1/v_l^3 + 2/v_t^3)/3$.

Section S3: Crystal structure, phononic structure and thermal properties

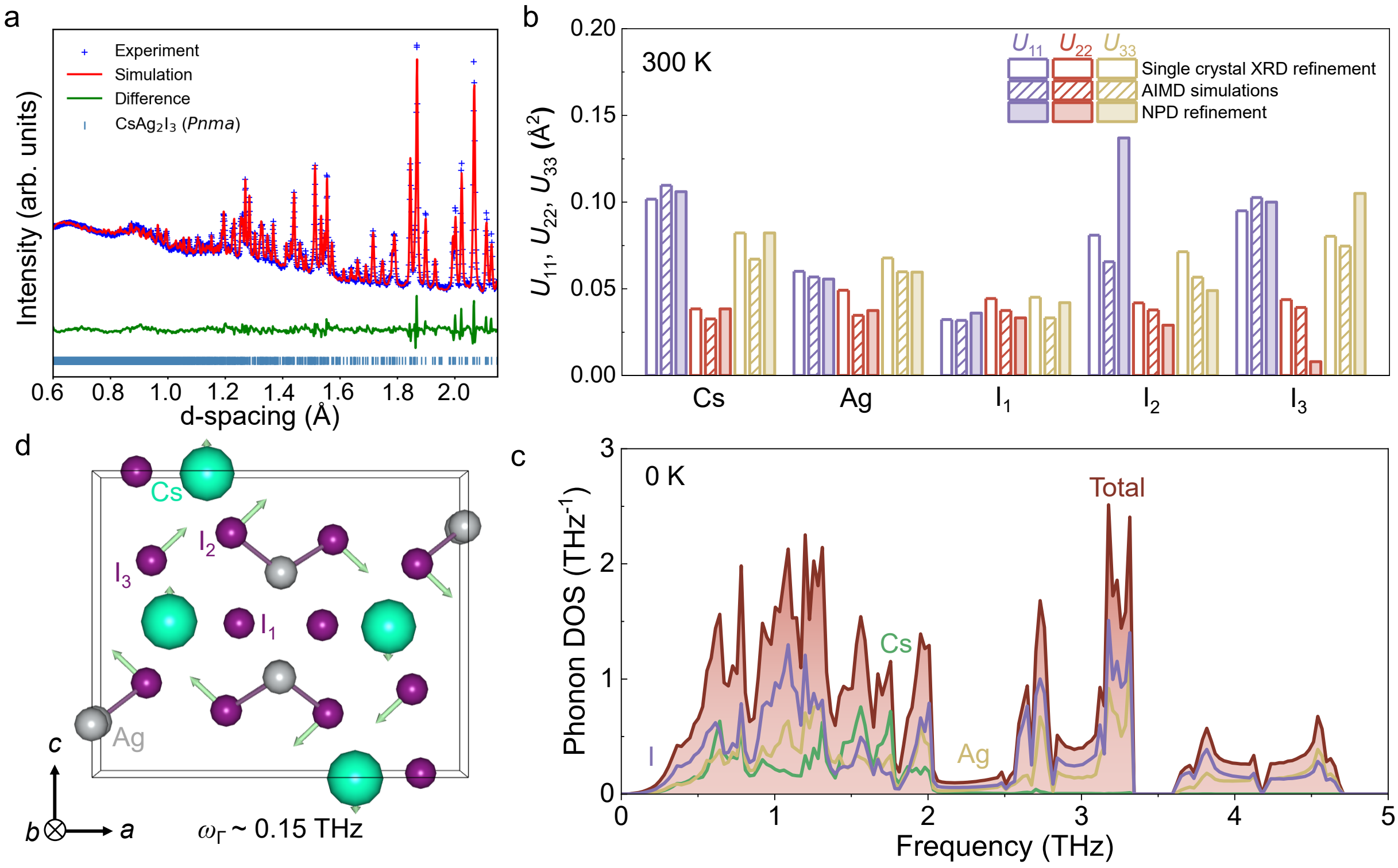

Figure S4. Structural dynamics and phonon properties. (a) Neutron powder diffraction Rietveld refinement at 300 K. (b) The diagonal mean-square displacements from Cs, Ag and I atoms at 300 K. (c) Computational element-resolved phonon density of states of $CsAg_2I_3$ at 0 K. (d) Vibration feature of 1st transverse optical mode with frequencies of 0.15 THz at the Γ point, viewed along the *b*-axis.

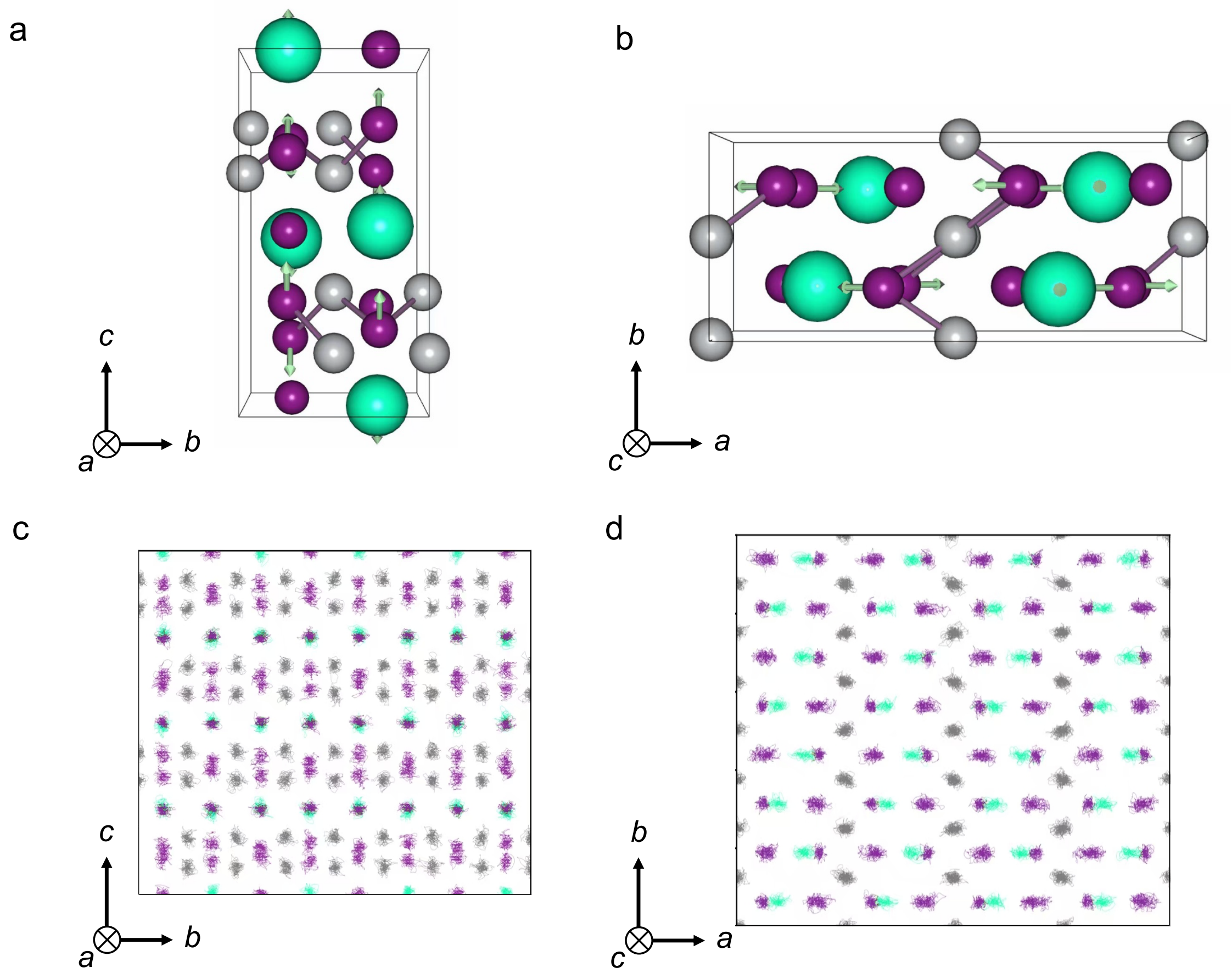

Figure S5. Vortical vibration modes and atomic trajectories in $CsAg_2I_3$. Top view along *a*- (a) and *c*- (b) axes of the vortical vibrations with frequencies of 0.15 THz at the Γ point in a unit cell. Room-temperature atomic trajectory projected onto the (100) plane (c) and (001) plane (d) in 2×4×2 supercell, with Cs in green, Ag in grey, and I in purple.

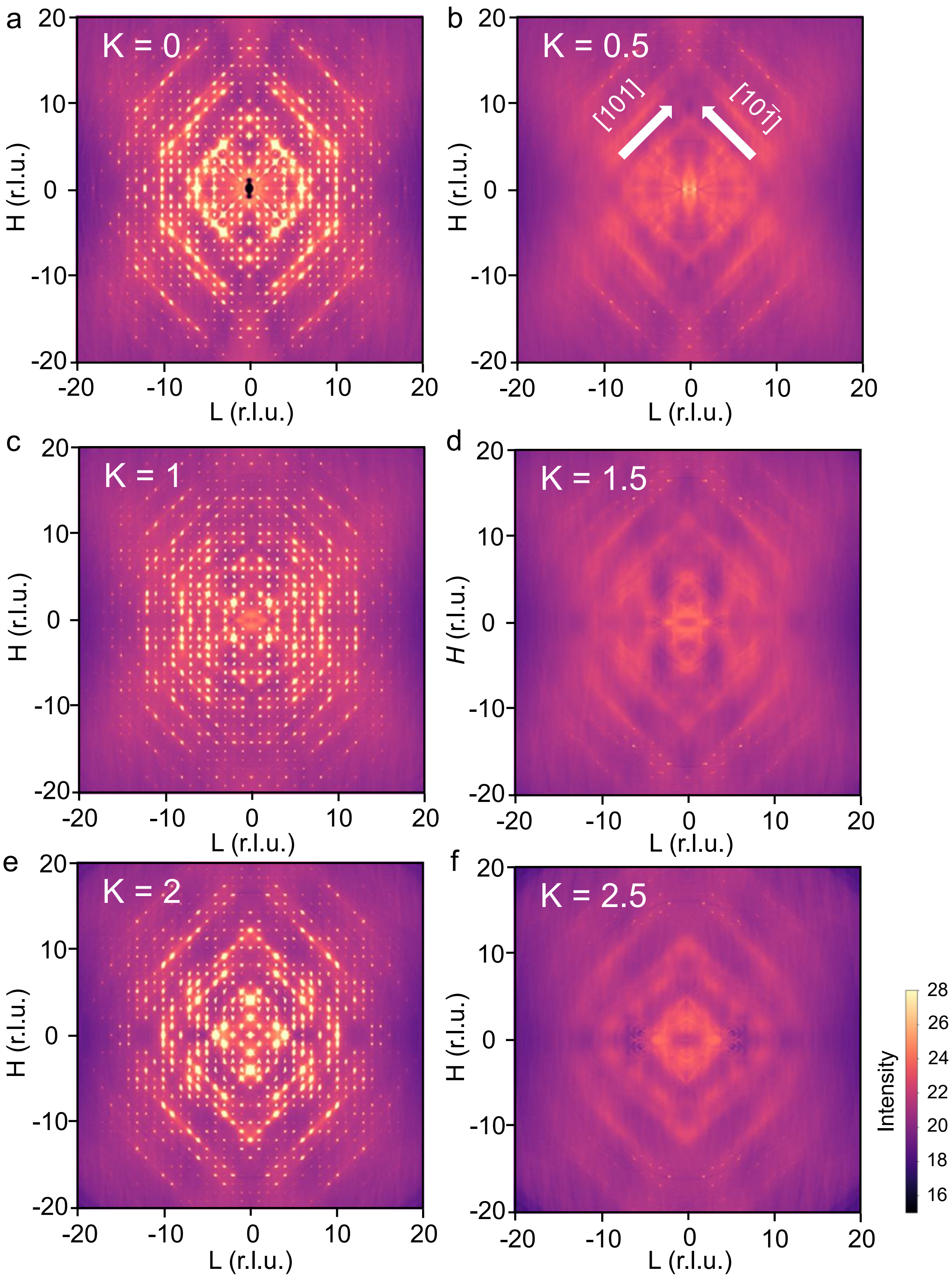

Figure S6. Room-temperature X-ray diffuse scattering of $CsAg_2I_3$ single crystal. Diffuse scattering patterns at the $Q = (HKL)$ planes, indicating the existence of short-range order. The white arrows mark the direction of the diffuse rods.

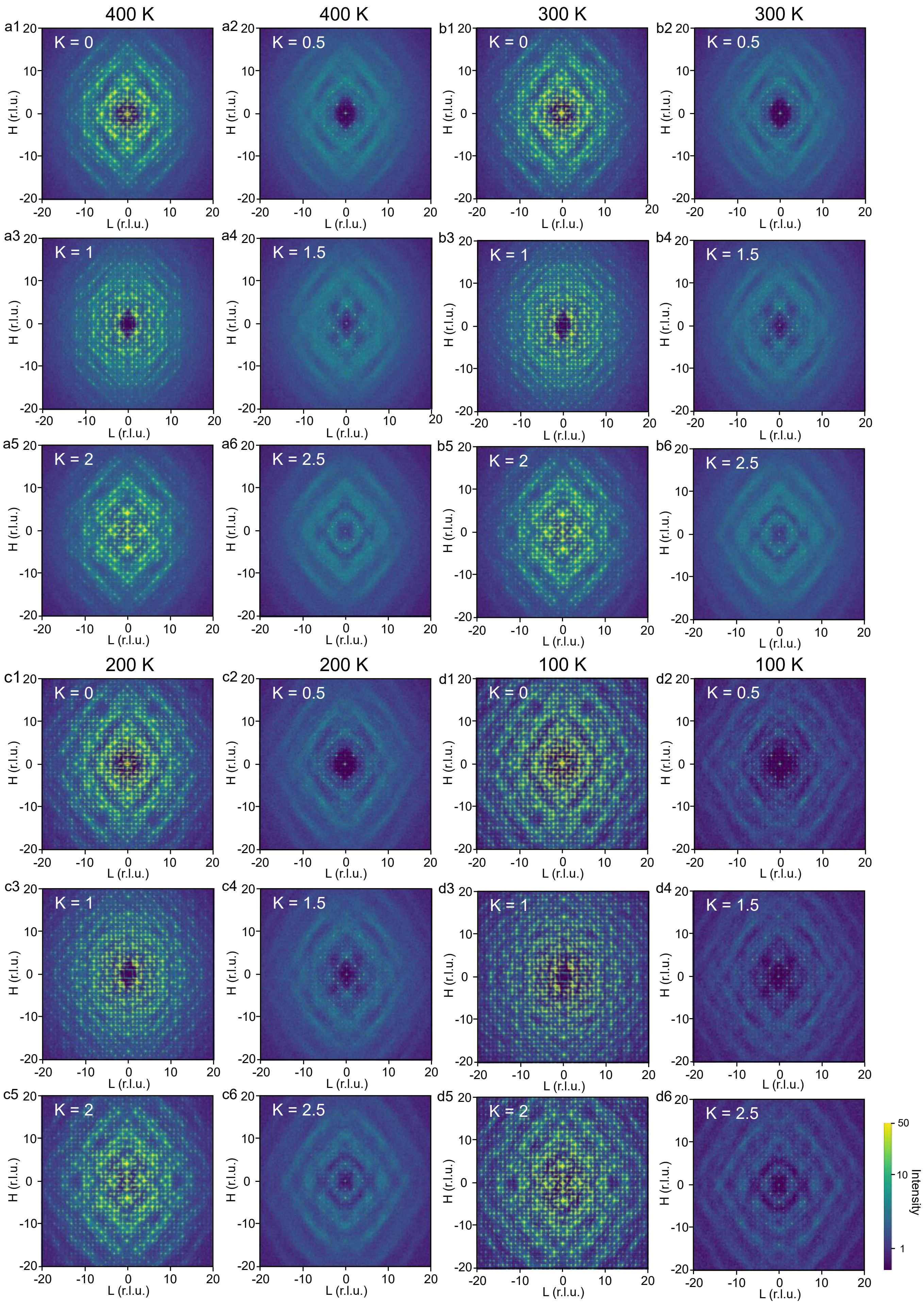

Figure S7. Simulated dynamic structure factor ($S(Q)$). Simulated $S(Q)$at the $Q = (HKL)$ planes of $CsAg_2I_3$, calculated from MD simulations at (a) 400 K, (b) 300 K, (c) 200 K, and (d) 100 K.

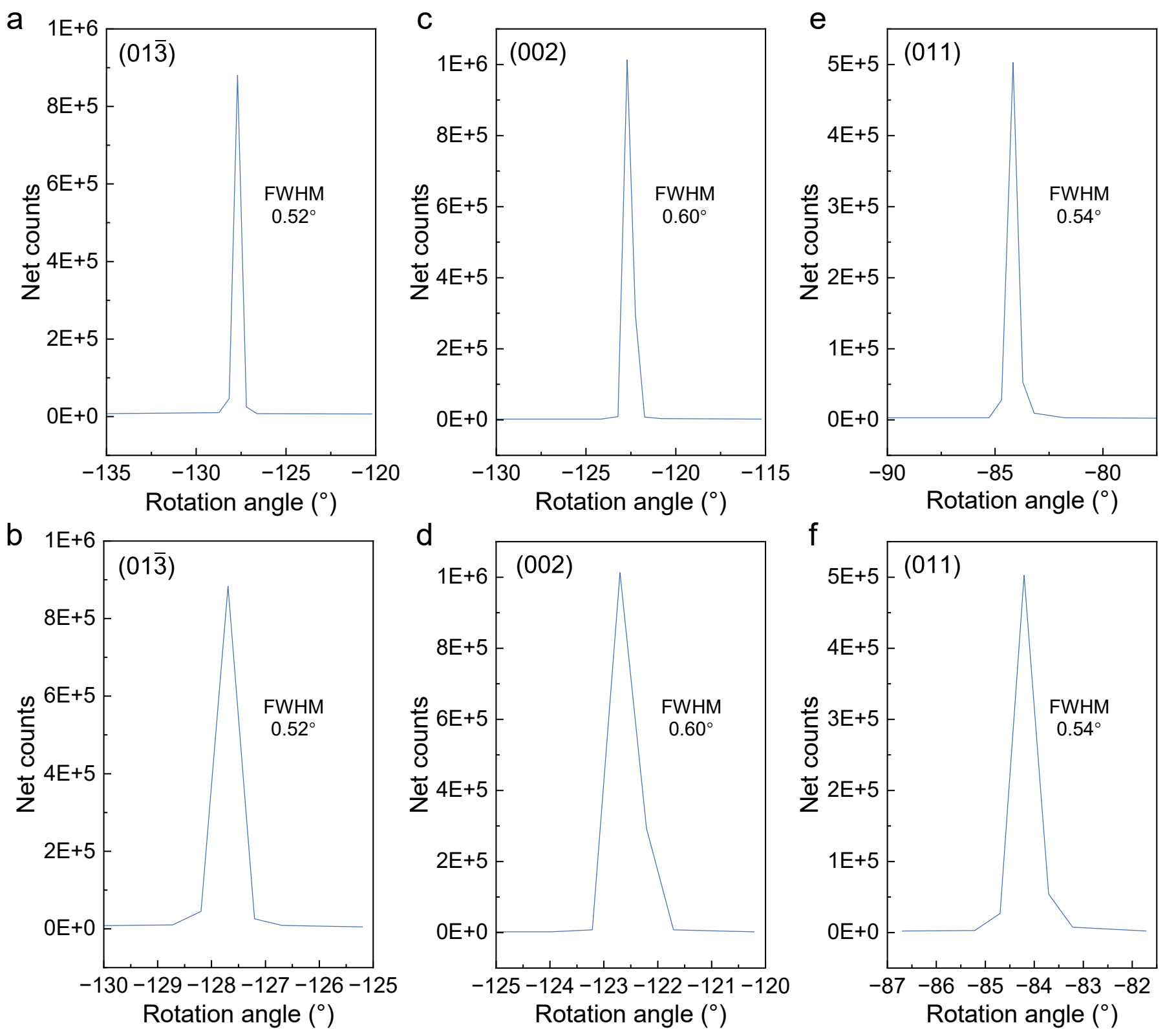

Figure S8. Rocking curves of $CsAg_2I_3$ single-crystal XRD. Rocking curves measured for the (01$\bar{3}$) (a-b), (002) (c-d), and (011) (e-f) lattice planes at 300 K.

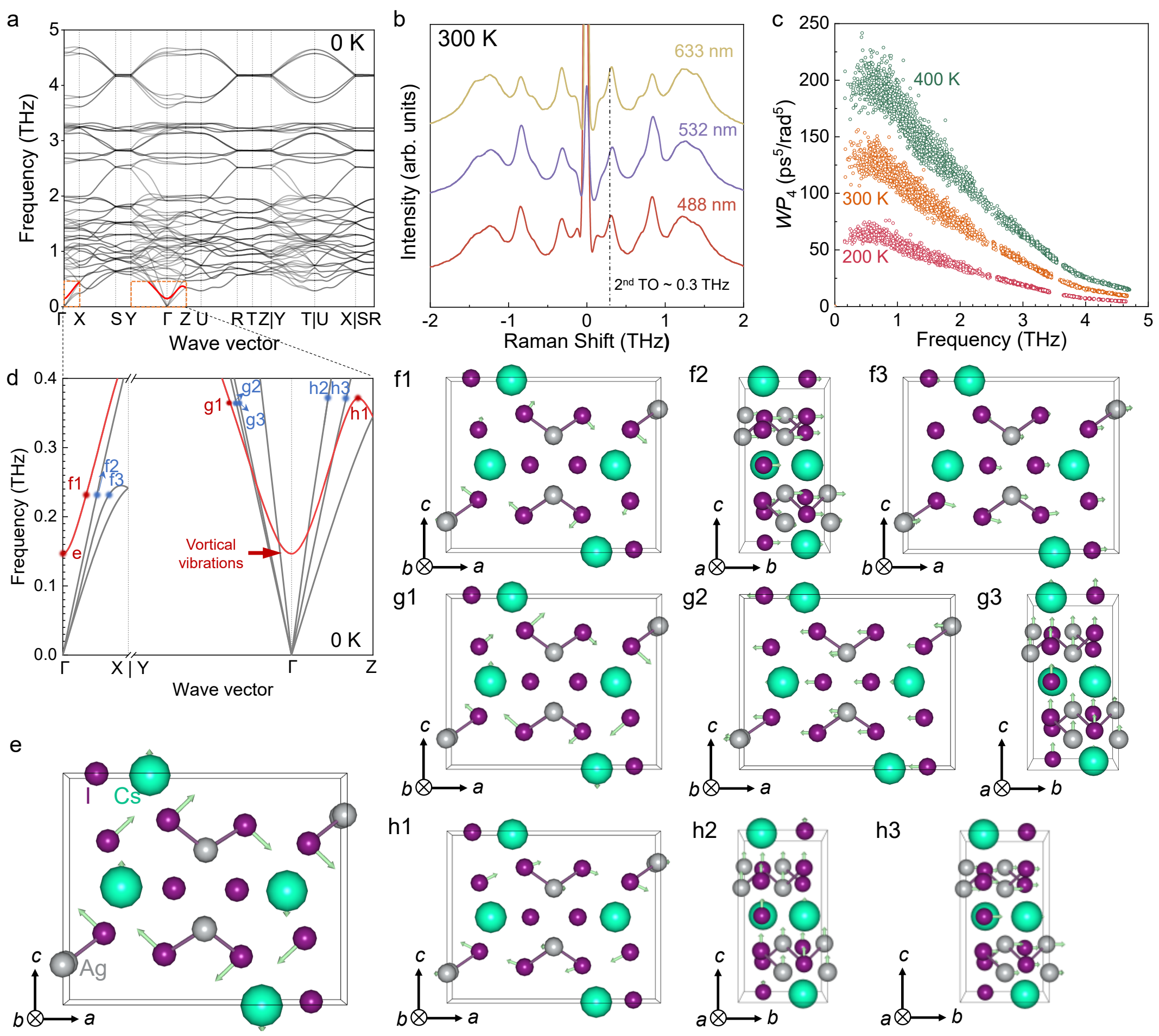

Figure S9. Phonon spectra and vibrational properties. Calculated phonon spectrum at 0 K (a) with the low-lying optical branch marked in red. (b) Raman spectra with different excitation wavelengths at room temperature. (c) The weighted four-phonon phase space (WP_4) at different temperatures. (d) First-principles calculated phonon spectrum at 0 K with the highlight of the characteristic vibration modes. (e-h) Parts of vibration features of $CsAg_2I_3$ along the paths of Γ to X, Γ to Y and Γ to Z.

Table S3. Computational Raman peaks of $CsAg_2I_3$ at 0 K.

Frequency (THz)	Irreducible representation	Intensity (Å^4/amu)	FWHM (cm^{-1})
0.14	A_g	0.012	1.50
0.50	B_{2g}	8.047	1.37
0.70	A_g	2.055	1.22
0.73	A_g	0.246	1.25
0.77	B_{3g}	0.0006	0.88
1.02	B_{1g}	0.006	1.59
1.03	B_{2g}	8.54	4.83
1.06	B_{2g}	0.29	1.59
1.07	B_{3g}	2.12	2.04
1.09	B_{1g}	28.6	2.99
1.20	B_{1g}	0.006	5.04
1.24	B_{2g}	0.008	2.37
1.25	A_g	25.8	3.31
1.28	B_{3g}	41.3	3.51
1.36	A_g	37.7	4.28
1.42	B_{2g}	0.095	3.86
1.43	B_{2g}	2.02	3.28
1.46	B_{1g}	0.84	2.83

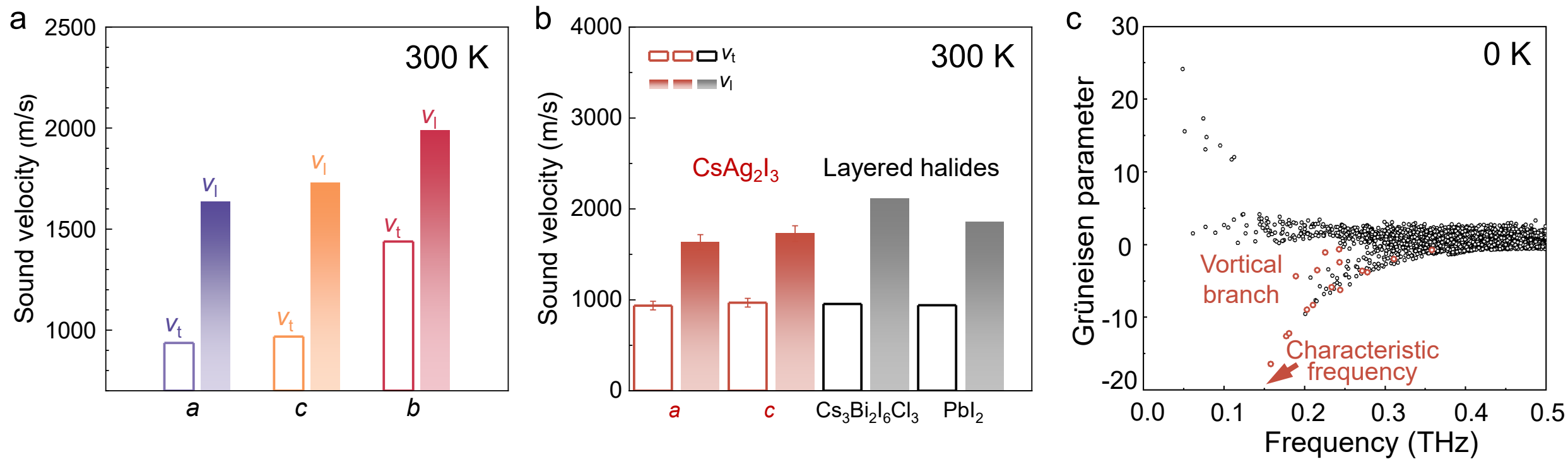

Figure S10. Sound velocities and Grüneisen parameter. (a) Measured transverse (v_t) and longitudinal (v_l) sound velocities along different crystal axes of $CsAg_2I_3$. (b) Room-temperature longitudinal and transversal sound velocities along the *a*- and *c*-axes of single crystalline $CsAg_2I_3$, as compared to those along the cross-plane direction of layered halide materials[2,3]. The error bars represent the propagated uncertainty originating from the measurement of sample thickness, corresponding to a relative uncertainty of approximately ±5%. (c) Calculated Grüneisen parameter of $CsAg_2I_3$ at 0 K.

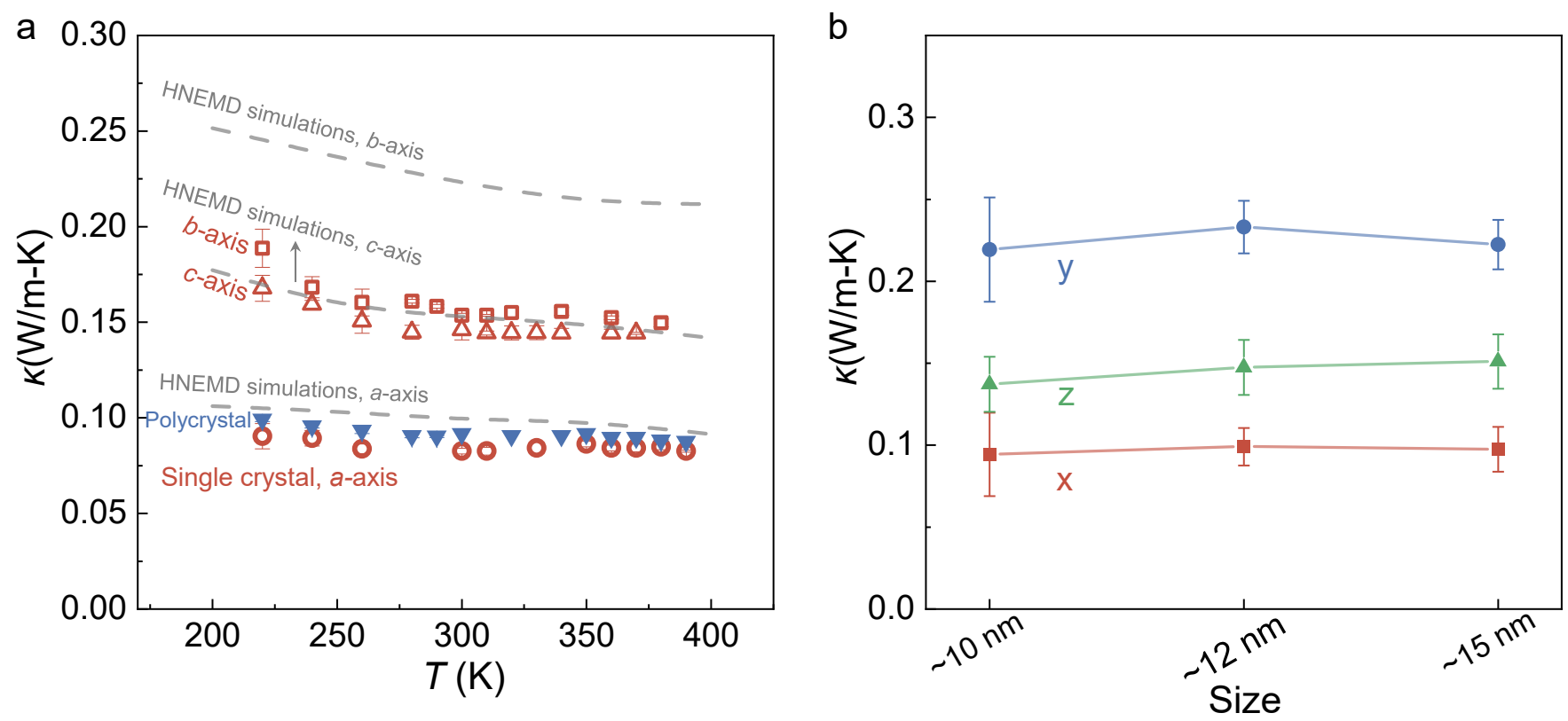

Figure S11. Thermal conductivity from experiment and HNEMD simulations. (a) Experimental and Homogeneous non-equilibrium molecular dynamics (HNEMD) simulated thermal conductivity along *a*-, *b*- and *c*-axes. The error bars represent the standard deviation (SD) derived from multiple independent measurements at each temperature, corresponding to a relative uncertainty of ±2–4%. (b) Convergence test of the cell size in the HNEMD simulations for computing lattice thermal conductivity of $CsAg_2I_3$. The error bars represent the standard deviation (SD) derived from 15 independent HNEMD simulations of the heat flux performed at each temperature, quantifying the statistical uncertainty of the averaged directional thermal conductivities.

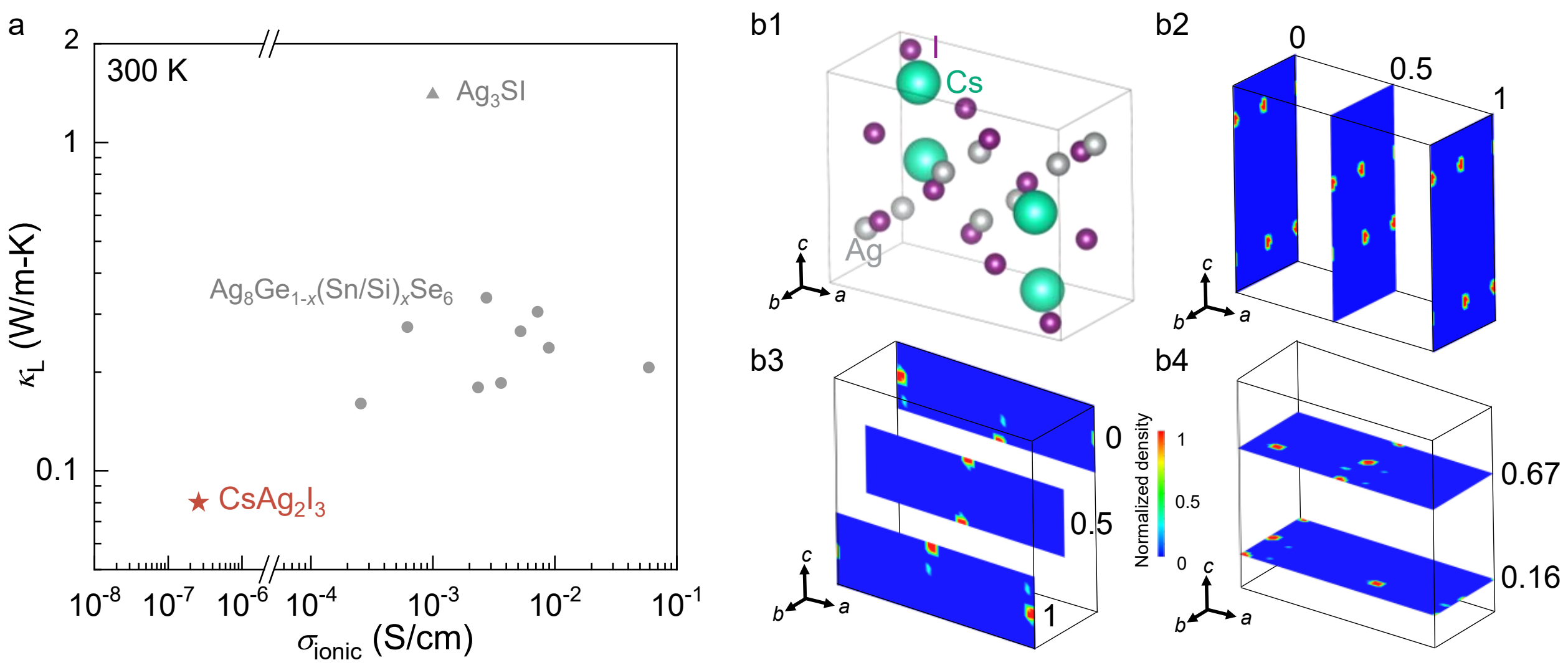

Figure S12. Ionic transport properties. (a) Lattice thermal conductivity vs. ionic conductivity for $CsAg_2I_3$ and superionic conductors[4,5] at 300 K. Schematic diagram of the cut surface in the crystal structure (b1) for the corresponding projection of electron densities of $CsAg_2I_3$ along the *a*-axis (b2), *b*-axis (b3) and *c*-axis (b4) visualized using VESTA.

The ionic conductivity was carried out by Chenhua 760 electrochemical workstation. For the maximum entropy method (MEM) density analysis, structure factors extracted from the powder X-ray diffraction Rietveld refinements were used. The MEM refinements were performed based on Dysnomia program[6,7]. The unit cell was divided into 56×32×48 pixels along the *a*, *b*, and *c* crystallographic axes and the calculation was initiated from a uniform prior density. Finally, the 3D electron density distribution and 2D slices were visualized by VESTA[8].

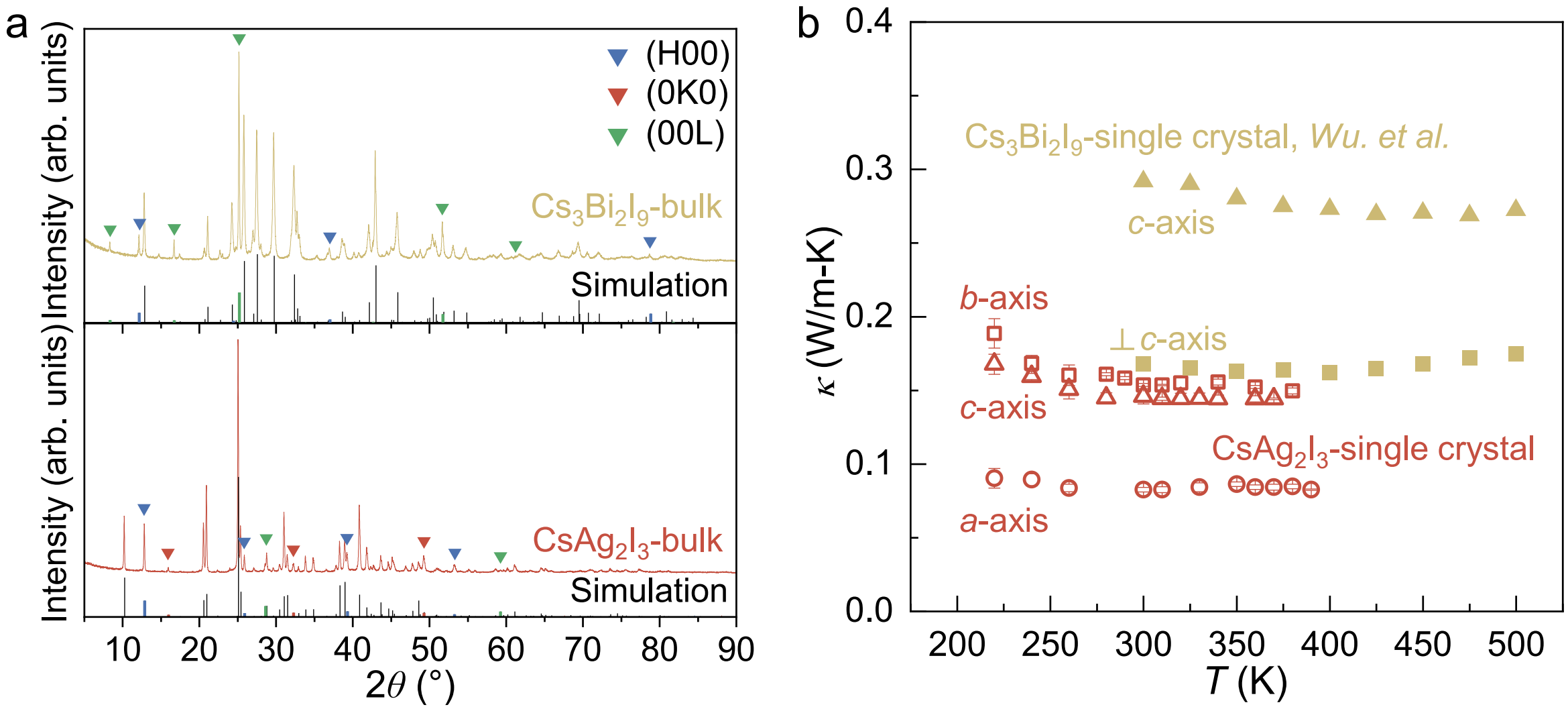

Figure S13. Structural characterization and thermal conductivity comparison of $CsAg_2I_3$ and $Cs_3Bi_2I_9$. (a) The XRD patterns of the $CsAg_2I_3$ and $Cs_3Bi_2I_9$ cylinders. (b) Experimental thermal conductivity of $CsAg_2I_3$ along the *a*-, *b*- and *c*-axes, and of $Cs_3Bi_2I_9$ parallel and perpendicular to the *c*-axis[9]. The error bars represent the standard deviation (SD) derived from multiple independent measurements at each temperature, corresponding to a relative uncertainty of ±2–4%.

Table S4. Orientation factor of (*h*00), (0*k*0) and (00*l*) lattice planes of $CsAg_2I_3$ and $Cs_3Bi_2I_9$.

**Materials**	***F*(*h*00)**	***F*(0*k*0)**	***F*(00*l*)**
$CsAg_2I_3$	0.037096	-0.01683	0.010151
$Cs_3Bi_2I_9$	-0.00747	/	0.13308

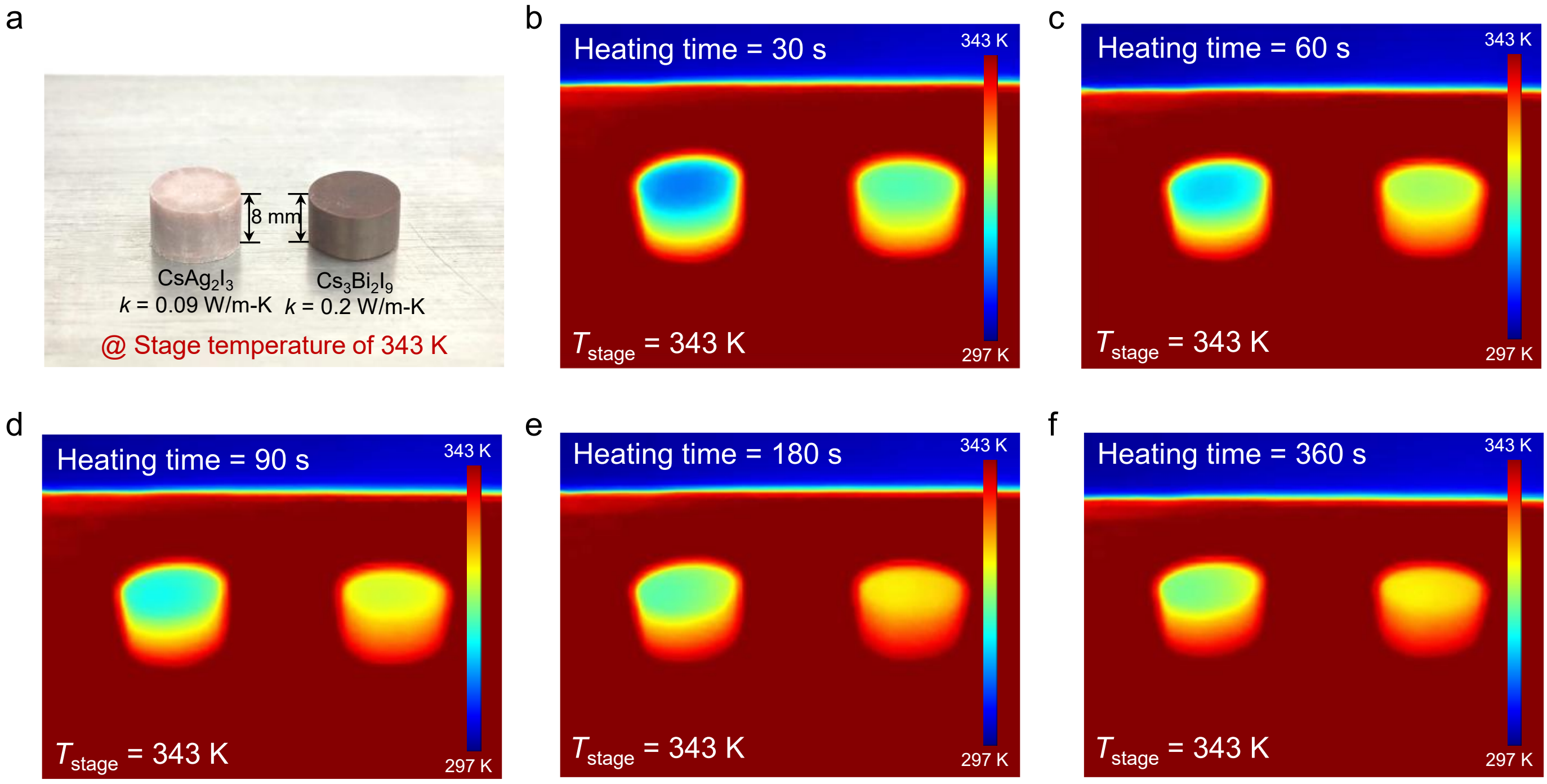

Figure S14. Time-dependent temperature distribution of $CsAg_2I_3$ and $Cs_3Bi_2I_9$. (a) Optical and (b–f) infrared camera images of $CsAg_2I_3$ and the layered halide materials $Cs_3Bi_2I_9$[9,10] placed on a 343 K hot stage, recorded over time.

Infrared photos were taken by HINMICRO K20.

Supplementary references

1 Kundu, R. Phonon spectrum in graphene. *arXiv*, 0710.2077, (2007).

2 Acharyya, P., Ghosh, T., Pal, K. *et al.* Glassy thermal conductivity in $Cs_3Bi_2I_6Cl_3$ single crystal. *Nat. Commun.* **13**, 5053, (2022).

3 Dorner, B., Ghosh, R. E. & Harbeke, G. Phonon dispersion in the layered compound PbI_2. *Physica Status Solidi-B-Basic Solid State Physics* **73**, 655-659, (1976).

4 Bernges, T., Hanus, R., Wankmiller, B. *et al.* Considering the role of ion transport in diffuson-dominated thermal conductivity. *Adv. Energy Mater.* **12**, 2200717, (2022).

5 Hull, S., Keen, D. A., Madden, P. A. *et al.* Ionic diffusion within the alpha* and beta phases of Ag_3SI. *J. Phys. Condens. Matter* **19**, 406214, (2007).

6 Momma, K., Ikeda, T., Belik, A. A. *et al.* Dysnomia, a computer program for maximum-entropy method (MEM) analysis and its performance in the MEM-based pattern fitting. *Powder Diffraction* **28**, 184-193, (2013).

7 Sakata, M. & Sato, M. Accurate structure analysis by the maximum-entropy method. *Acta Crystallogr.* **A46**, 263-270, (1990).

8 Momma, K. & Izumi, F. VESTA 3 for three-dimensional visualization of crystal, volumetric and morphology data. *Journal of Applied Crystallography* **44**, 1272-1276, (2011).

9 Wu, L., Chen, Z., Bai, Q. *et al.* Geometric configuration of polyhedrons dominating particle- to wave-like thermal transport in single crystalline $Cs_3Bi_2I_9$. *National Science Open* **4**, 20250056, (2025).

10 Acharyya, P., Pal, K., Ahad, A. *et al.* Extended antibonding states and phonon localization induce ultralow thermal conductivity in low dimensional metal halide. *Adv. Funct. Mater.* **33**, 2304607, (2023).
